# Dietary Compound Isoliquiritigenin Inhibits Breast Cancer Neoangiogenesis *via* VEGF/VEGFR-2 Signaling Pathway

**DOI:** 10.1371/journal.pone.0068566

**Published:** 2013-07-05

**Authors:** Zhiyu Wang, Neng Wang, Shouwei Han, Dongmei Wang, Suilin Mo, Linzhong Yu, Hui Huang, Kamchuen Tsui, Jiangang Shen, Jianping Chen

**Affiliations:** 1 School of Chinese Medicine, The University of Hong Kong, Pokfulam, Hong Kong, China; 2 Guangdong Provincial Hospital of Chinese Medicine, The Second Clinical Medical Collage, University of Guangzhou Traditional Chinese Medicine, Guangzhou, China; 3 School of Pharmaceutical Sciences, Sun Yat-sen University, Guangzhou, China; 4 The First Affiliated Hospital, Sun Yat-sen University, Guangzhou, China; 5 School of Chinese Medicine, South Medical University, Guangzhou, China; 6 Sun Yat-sen Memorial Hospital, Sun Yat-sen University, Guangzhou, China; 7 The Hong Kong Associate of Chinese Medicine, Hong Kong, China; Duke University Medical Center, United States of America

## Abstract

Angiogenesis is crucial for cancer initiation, development and metastasis. Identifying natural botanicals targeting angiogenesis has been paid much attention for drug discovery in recent years, with the advantage of increased safety. Isoliquiritigenin (ISL) is a dietary chalcone-type flavonoid with various anti-cancer activities. However, little is known about the anti-angiogenic activity of isoliquiritigenin and its underlying mechanisms. Herein, we found that ISL significantly inhibited the VEGF-induced proliferation of human umbilical vein endothelial cells (HUVECs) at non-toxic concentration. A series of angiogenesis processes including tube formation, invasion and migration abilities of HUVECs were also interrupted by ISL *in vitro*. Furthermore, ISL suppressed sprout formation from VEGF-treated aortic rings in an ex-vivo model. Molecular mechanisms study demonstrated that ISL could significantly inhibit VEGF expression in breast cancer cells *via* promoting HIF-1α (Hypoxia inducible factor-1α) proteasome degradation and directly interacted with VEGFR-2 to block its kinase activity. *In vivo* studies further showed that ISL administration could inhibit breast cancer growth and neoangiogenesis accompanying with suppressed VEGF/VEGFR-2 signaling, elevated apoptosis ratio and little toxicity effects. Molecular docking simulation indicated that ISL could stably form hydrogen bonds and aromatic interactions within the ATP-binding region of VEGFR-2. Taken together, our study shed light on the potential application of ISL as a novel natural inhibitor for cancer angiogenesis *via* the VEGF/VEGFR-2 pathway. Future studies of ISL for chemoprevention or chemosensitization against breast cancer are thus warranted.

## Introduction

Neo-angiogenesis has been well demonstrated as a critical step in tumor growth, migration and metastasis. The neovasculature in a tumor mass not only supplies oxygen, nutrients and growth factors for tumor growth, but also provides vessels for tumor cell infiltration and migration. Tumors lacking an adequate vasculature become necrotic or apoptotic, while tumors with abundant vasculatures may not only enter a phase of rapid growth but also exhibit increased metastatic potential [1]. Thus, inhibiting angiogenesis has become an important strategy in cancer treatment.

Tumor angiogenesis is a complex process and involves the interaction between tumor cells, endothelial cells, phagocytes and their secreted factors, which may act as stimulators or inhibitors of angiogenesis [2], [3]. One of the initial events of angiogenesis is the secretion of multiple angiogenic factors from cancer cells, such as VEGF, bFGF and PDGF, etc [3], [4]. At present, VEGF has been identified as the most important pro-angiogenic factor [5], [6]. After binding with VEGF receptors on the surface of endothelial cell, signal pathways including Ras/Raf/MEK/ERK and PI3K/Akt will be activated, which sequentially promote endothelial cells recruitment and proliferation [7]–[9].

The human VEGF kinase receptors include VEGFR-1, VEGFR-2 and VEGFR-3. VEGFR-1 is required for the recruitment of bone marrow-derived progenitor cells and the migration of monocytes and macrophages, while VEGFR-3 is mainly reported to participate in lymphangiogenesis [10]. VEGFR-2 is the predominant mediator of VEGF-induced angiogenic signaling and is responsible for regulating vascular cells proliferation, migration and invasion [11]. VEGFR-2 null animals are reported to be embryonic lethal, characterized by endothelial cells not forming a structured, organized vascular network [12]. VEGFR-2 consists of 3 domains: the extracellular VEGF-binding domain, the transmembrane domain, and the intracellular catalytic domain possessing tyrosine-kinase activity. Upon binding to VEGF, the immunoglobulin-like segments in the extracellular domain will undergo dimerization, and then induce autophosphorylation within the intracellular catalytic domain by consuming ATP. The predominant phosphorylation sites on VEGFR-2 occur on tyrosine 1175 and 1214, inducing signaling cascades through PI3K, AKT, PLCγ, p38MAPK and p42/44 MAPK [13]. ATP-binding region located within the catalytic domain is most critical for VEGFR-2 activation. Most of current anti-angiogenesis inhibitors approved for clinical application are designed targeting on ATP-binding site such as sorafenib. However, serious side effects, such as hypertension, bleeding and gastrointestinal perforation, have been associated with long-term application of current anti-angiogenesis agents, and therefore limiting their chronic use [14]. Since natural extracts are usually low in toxicity and well tolerated in human body, there has a growing interest in identifying natural phytochemicals potentially efficient for anti-angiogenesis with less toxic effects [15].

Isoliquiritigenin (ISL) is a natural flavonoid isolated from the root of licorice (*Glycyrrhiza uralensis*), which is commonly used in Western countries for culinary purpose, while in China it is a medicinal herb. Previous research has demonstrated that ISL possessing various biologic properties, such as anti-inflammation, anti-oxidation, anti-platelet aggregation, as well as vasorelaxant and estrogenic effects [16]. In addition, a number of studies reported that ISL had significant antitumor activities, including apoptosis induction, cell cycle arrest, migration inhibition and oxidative stress triggering, etc [17]–[19]. Some studies also indicated that ISL was capable to mediate chemopreventive activities, including suppressing 7,12-dimethylbenz [a] anthracene (DMBA)-induced mouse skin carcinogenesis and the inhibition of carcinogen-induced lesion formation in a mouse mammary organ culture assay [20], [21]. Very recently, PHY906, a four-herb Chinese medicine formula with licorice as a major ingredient, was shown to reduce chemotherapy-induced toxicity in a phase I/II clinical study [22]. For cancer angiogenesis, a high throughput screening assay found that ISL had a higher efficacy in suppression of endothelial cell growth and migration when compared with other herbal chemicals and Avastin at a subtoxic concentration of 10 µM [23], [24]. From the chemical structure-activity prediction, ISL could have a greater antiangiogenic potency than other licorice-derived flavonoids (such as isoliquititin, liquiritigenin and isoliquiritin apioside) [25]. However, how ISL inhibits cancer neoangiogenesis and whether or not ISL still possess *in vivo* anti-neoangiogenesis effects are still remained unclear.

In the present study, the effects of ISL on inhibiting breast cancer angiogenesis were validated both *in vitro* and *in vivo*. Mechanism study revealed that ISL could significantly inhibit VEGF expression *via* promoting HIF-1α proteasome degradation pathway. Meanwhile, ISL could block VEGFR-2 activation and the transduction of its downstream signalings. *In silico* analysis further revealed that ISL suppressed VEGFR-2 activity *via* stably binding to its ATP binding site. Taken together, we suggest that ISL might be utilized to target angiogenesis in breast cancer treatment and chemoprevention as well as other angiogenic diseases.

## Materials and Methods

### Chemicals and Reagents

All animal work was approved by the Committee on the ethics of the University of Hong Kong (Permit number:2162-10). ISL was isolated from licorice by Dr. Wang Dongmei (Sun Yatsen University, Guangzhou, China). The standard of ISL was bought from Alpha Aesar company with a purity more than 97%. The purity of isolated ISL was more than 99% as analyzed by high performance liquid chromatography and its chemical structure was characterized by LC-MS and NMR. The stock solution of ISL was prepared in dimethyl sulphoxide (DMSO) and kept at −20°C. ISL was diluted in culture medium to obtain the desired concentration. ISL was stable in the dilution with DMSO concentration less than 1‰.

### Antibodies and other Materials

Recombinant VEGF-A_165_ was obtained from PeproTech Company (PeproTech, Rockyhill, NJ). Endothelial cell growth supplement (ECGS) and Matrigel was obtained from BD Bioscience Company (BD bioscience, Bedford, MA). BrdU labeling kit, Lactate dehydrogenase (LDH) cytotoxicity kit, the first strand CDNA synthesis kit, the SYBR Green kit and TUNEL kit were bought from Roche Company (Roche Diagnostics, IN). Human VEGF Quantikine ELISA Kit was obtained from R&D Systems Company (R&D Systems, Minneapolis, MN). The enzyme-linked immunosorbent assay kit was bought from Boehringer Mannheim Company (Boehringer Mannheim, SA). The TRIzol reagent, Co-IP assay kit and primers were ordered from Invitrogen (Invitrogen, Carlsbad, CA). The immunohistochemistry kit was obtained from Thermo Scientific Company (Thermo scientific, Fremont, CA). All primary and secondary antibodies were bought from Cell Signaling Company (Cell Signaling Technology, Danvers, MA).

### Cell Culture

Human breast cancer cell lines MCF-7 and MDA-MB-231 were obtained from the American Type Culture Collection and cultured in medium (DMEM for MCF-7; L-15 for MDA-MB-231) supplemented with 10% FBS and 1% penicillin and streptomycin at 37°C in a humidified incubator. Human umbilical endothelial cell line was also bought from American Type Culture Collection and was grown on a 0.2% gelatin-coated flask and cultured with M199 medium supplemented with 100 µg/ml ECGS, 15% FBS and 0.1% heparin. HUVECs between passages 2 and 6 were used in our experiments. No further authentication was conducted for cell lines.

### Proliferation Assay

HUVECs were seeded in a 6-well plate with a density of 1×10^5^ cells/well. After being left overnight, cells were incubated in serum-starved M199 for 12 h, and then treated with VEGF (20 ng/ml) in the presence or absence of ISLfor 48 h. After incubation, the cell number was counted by trypan blue staining. For proliferation analysis, the BrdU labeling solution was added and the cells were incubated in 96 well plates (4×10^3^ cells/well) for 12 h. After the incubation, the effect of ISL on VEGF induced proliferation of the HUVECs was determined by the extent of BrdU incorporation using the protocol supplied by the manufacturer. Briefly, after the treatment of BrdU labeling solution, the medium was aspirated and the cells were fixed and incubated with anti-BrdU antibody. After washing three times, the cells were incubated with the secondary antibody conjugated with horse radish peroxidase. Finally, the extent of BrdU incorporation was determined colorimetrically at 450 nm. Triplicate independent experiments were conducted. For non-endothelial cell proliferation, MCF-7 and MDA-MB-231 (1×10^5^ cells/well) were plated onto 6-well plates and then treated with various concentrations of ISL. Cell numbers were counted after 48 h incubation. For cell cycle analysis, HUVECs (3×10^5^/well) were synchronized in G1 phase by serum deprivation then treated by ISL. Cells were harvested after 24 h. Propidium iodide-stained single-cell suspension was analyzed on FACs Calibur (BD Bioscience) using CellQuest and ModFit data analysis software for data analysis.

### Lactate Dehydrogenase (LDH) Cytotoxicity Assay

The LDH released into cell cultures is an index of cytotoxicity and evaluation of the permeability of cell membrane. HUVECs were seeded in 96-well plates at a density of 5×10^3^/well. After incubation with various concentrations of ISL for 48 h, cell supernatants were collected and analyzed for LDH activity using a LDH cytotoxicity assay kit. The absorbance of formed formazan was read at 490 nm on a microplate reader.

### *In vitro* Tube Formation Assay

The tube formation assay was performed using 24-well plates coated with 100 µl Matrigel basement membrane matrix per well and polymerized at 37°C for 30 min. HUVECs suspended in M199 medium containing 2% FBS were plated on the Matrigel at a density of 1×10^5^ cells/well. Different concentrations of ISL were then added to the well. After 12 h, cells were photographed with a digital camera attached to an inverted microscope. Triplicate independent experiments were conducted.

### Cell Invasion and Wound Healing Assay

The invasion assay was performed using transwell inserts containing 8 um pore size filters (Corning, NY, USA). Briefly, the upper and lower parts of the transwell inserts were coated with 20 µl Matrigel and 40 µl type I collagen (0.5 mg/ml), respectively. HUVECs (5×10^5^ cells/ml) were seeded to each insert (upper chamber), and the chemo-attractant (10% FBS) was placed in the lower chamber. Different concentrations of ISL were then added to the upper chamber. After 24 h, the upper surface of the transwell was wiped out with a cotton swab to remove the remaining cells. The cells in the lower surface of the membrane were fixed with 4% paraformaldehyde and stained with the HE method. Migration was normalized to percent migration, with migration in the presence of VEGF representing the scale of 100%. For wound healing assay, After HUVECs reached over 90% confluency in 6-well plates, a scrape was made over cells by a 10 µl tip. After scraping, the cells were washed with PBS twice and treated by vehicle or ISL. The migration inhibition activity of ISL was evaluated by measuring the gap width between cells after 24 h. Triplicate independent experiments were conducted.

### Chick Aortic Ring Assay

The aortic arch was dissected from day 12–14 chick embryos. After cutting into rings, they were embedded into Matrigel in 24-well plates. After incubation for 10 mins at 37°C, the aortic rings were supplemented with M199 serum-free medium containing various concentrations of ISL. Sprouts will be formed within 48–72 hrs. Images were photographed at 5× objective of a Zeiss inverted microscope at 50×magnification. The extent of sprouts formation from chick aortic ring was quantified using image-pro software.

### Quantification of VEGF

For the measurement of VEGF production, MCF-7 and MDA-MB-231 cells were cultured in serum-free medium for 48 hours in the absence or the presence of ISL. Cell-free culture supernatants were harvested and used for the determination of VEGF levels by Elisa method. The concentration of VEGF in the unknown samples was then determined by comparing the optical density of the samples to the standard curve. To determine VEGF mRNA levels, total RNA in both cancer cells after ISL treatment were extracted using TRIzol reagent and reverse transcription were carried out using first strand CDNA synthesis kit according to the manufacturer’s instruction. qPCR analysis was performed using a SYBR Green on Roche lightcycler 480 detector. The primers for VEGF and β-actin were designed as followings: VEGF, forward primer: 5′-TGCCCGCTG CTGTC TAAT-3′, reverse primer: 5′-TCTCCGCTCTGA GCAAGG-3′; β-actin: forward primer: 5′-CCAACCGCGAGAAGA TGA-3′, reverse primer: 5′-CCAG AGGCG TACAGGGATAG-3′. The cycling parameters were 95°C for 15 seconds, 60°C for 30 seconds, and 72°C for 30 seconds for 40 cycles, followed by a melting curve analysis. Ct value was measured during the exponential amplification phase. The relative expression level (defined as fold change) of target gene is given by 2^−ΔΔCt^ and normalized to the fold change detected in the corresponding control cells, which was defined as 1.0.

### *In vitro* Kinase Assay and Immunoblotting Analysis

*In vitro* VEGFR-2 tyrosine kinase activity was assayed using an enzyme-linked immunosorbent assay kit. Briefly, ISL was incubated with biotin-labeled VEGFR-2 (Prospec) in assay buffer containing Mg^2+^ and ATP in 96-well plates coated with a streptavidin. Phosphorylated tyrosine was then detected by sequential incubation with a mouse IgG anti-phosphotyrosine antibody and a HRP-linked sheep anti-mouse immunoglobulin antibody. Color was developed with an HRP chromogenic substrate and quantified by an ELISA reader at wavelength 450 nm. The results were expressed as percent kinase activity. For Western blotting analysis, quantified protein lysates (30 µg) were resolved on SDS-PAGE gel, transferred onto PVDF membrane (Millipore, Billerica, MA), and immunoblotted with antibodies for HIF-1α, ERK1/2, JNK, Akt, VEGFR2, eNOS and β-actin. Triplicate independent experiments were conducted.

### Immunoprecipitation Assay

For immunoprecipitation assay, Immunoprecipitation kit-Dynabeads Protein G was applied. Briefly, control or drug treated cancer cells were lysed in RIPA buffer. Followed by centrifugation, the supernatants were collected and incubated with protein G dynabeads, which was binding to antibody (VEGFR-2) in advance. After incubation at room temperature for 2 h, the Dynabeads-Ab-Ag complex were washed three times with provided washing buffer and denaturized for following immuoblotting experiments with VEGF and VEGFR-2.

### Gelatin Zymography

Supernatants from a HUVEC culture system in the presence or absence of ISL were analyzed for gelatin degradation activity by sodium dodecyl sulfate-polyacrylamide gel electrophoresis under non-reducing conditions. One milligram per milliliter of gelatin was prepolymerized on a 10% polyacrylamide gel as a substrate. Electrophoresis was carried out at 4°C. The gel was washed with washing buffer (50 mM Tris-HCl, PH7.5, 100 mM NaCl and 2.5% Triton X-100), followed by incubation with a buffer (50 mM Tris-HCl, PH 7.5, 150 mM NaCl, 10 mM CaCl_2_, 0.02% NaN_3_ and 1 uM ZnCl_2_) at 37°C for 16 h and visualized with Coomassie Blue R-250.

### *In vivo* Tumor Xenograft Experiment

MDA-MB-231 cells were injected into the mammary gland of a 4–6 week old female nude mouse. After reaching approximately 1 cm^3^, tumors were obtained and cut into 5 mm^3^ the pieces were then transplanted to mammary glands. Mice were divided into vehicle (PBS) and drug treated groups (6 mice per group). ISL was given by intraperitoneal injection at a concentration of 25 mg/kg/d and 50 mg/kg/d. Tumor size was measured every three days and tumor volume was calculated according to a standard formula: (mm^3^) = L ×W ^2^/2, where L is the length and W is the width. Tumor-bearing mice were euthanized after 25 days of treatment and tumor weight was recorded. Body weight was also monitored weekly as an indicator of overall health of the animals.

### Immunohistochemistry

Tumor samples obtained from *in vivo* studies were rinsed in PBS and fixed in 10% paraformaldehyde/PBS. Samples were dehydrated in 70% ethanol, paraffin embedded, and sectioned (4 um). Deparaffinized sections were stained for CD31, p-VEGFR2, P-ERK1/2 and MMP2 antigen. Briefly, samples were treated with 3% H_2_O_2_ at -20°C for 10 min to neutralize endogenous peroxidase activity. Sections were then blocked in 5% BSA and incubated with primary antibodies at 4°C overnight in a humid chamber, followed by biotinylated secondary antibodies. Detection was done with avidin-biotin-HRP complex (Thermo scientific, Fremont, CA) and di-aminobenzidine as chromogen. Nuclei were counterstained with hematoxylin. For TUNEL assay, deparaffinized sections were permeabilized with 0.1% Trition X-100 plus 0.1% sodium citrate and then incubated with 50 µl TUNEL reaction mixture at 37°C for 60 mins. After rinsing with PBS three times, 50 ul Converter-POD was added and the tissue cells were incubated in a humidified chamber for 30 min at 37°C. DAB substrate was then added, followed by counterstaining with hematoxylin. Antigen-positive cells were counted in six fields per tumor sample and analyzed by Image-pro analysis.

### *In silico* Analysis

The CDOCKER module in Discovery studio (DS) 2.1 was selected as our molecular docking algorithm. Chemoffice 2002 (Cambridgesoft, Cambridge, MA) was used to draw the chemical structure of ISL. The three dimensional (3D) crystal structure of VEGFR2 was determined from PDB (http://www.rcsb.org/pdb/) with the ID of 3VHE [26]. Accuracy testing was performed by calculating the root mean square deviation (RMSD) after docking the internal ligand (Compound 20d:1-{2-fluoro-4-[(5-methyl-5H-pyrrolo[3,2-d]pyrimidin-4-yl)oxy]phenyl}-3-[3 -(tri fluoromethyl)phenyl]urea) with the algorithm into the crystal structure of VEGFR2. The water molecules in the picture of single crystal diffraction of VEGFR2 were removed, and finally the protein was refined with CHARMM in DS 2.1. For the docking purpose, the ATP binding site within VEGFR-2 was defined as the ligand-binding site, and ISL was docked into VEGFR-2 with proper parameter setting. Specifically, starting from the initial configuration, 100 different orientations of ISL were randomly generated and docked into the ATP pocket of VEGFR-2. A molecular dynamic simulation would be then carried out consisting of a heating phase from 300 to 700 K with 2,000 steps and a cooling phase back to 300 K with 5,000 steps. The binding energy was calculated as a score to rank the docking poses. The top 10 docking poses will be finally saved.

### Data Analysis

The data were expressed as mean ± SD. A two-tailed Student’s t-test was used to examine the significance of the data. Statistical significance was considered when the *P* value <0.05.

## Results

### ISL Inhibits the Proliferation of Endothelial Cells Induced by VEGF

**Figure 1:**
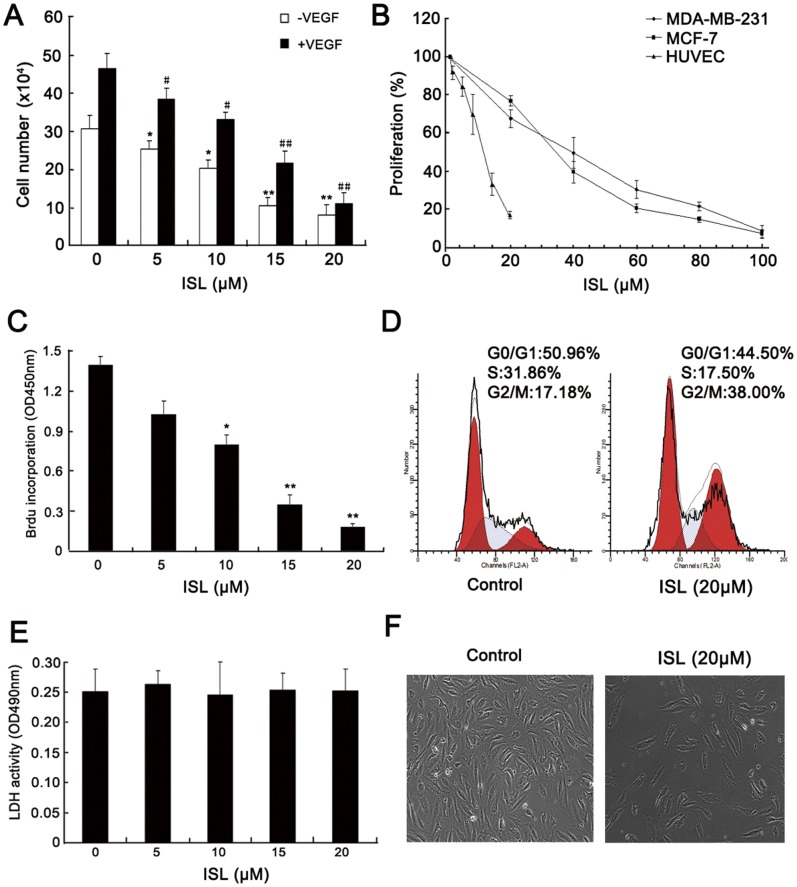
ISL inhibited endothelial cells proliferation. (A) After incubation with various concentrations of ISL for 48 h with and without VEGF stimulation, the number of endothelial cells were counted. The results showed that the HUVEC proliferation was inhibited by ISL in a dose-dependent manner; (B) Breast cancer cells MDA-MB-231, MCF-7 and endothelial cells were treated with various concentrations of ISL and cells were counted after 48 h. ISL exhibited a specific proliferation inhibition effect on endothelial cells in comparison with that of breast cancer cells. Data are represented as a percentage of the vehicle-treated control; (C) Endothelial cells were treated with various concentrations of ISL plus with VEGF stimulation for 48 h and labeling with BrdU. DNA synthesis was measured by enzyme-linked immunosorbent assay. ISL significantly suppressed DNA synthesis of endothelial cells in a dose-dependent manner; (D) Cell cycle analysis revealed that after ISL (20 µM) treatment for 48 h, the cell cycle of HUVECs was significantly arrested at G2/M checkpoint; (E) Endothelial cells culture supernatants after ISL treatment were collected and analyzed for LDH activity assay. The results showed that ISL administration did not lead to LDH release from cells, indicating that ISL has little cytotoxicty effect on endothelial cells; (F) After ISL (20 µM) administration for 48 h, there has little morphological changes of endothelial cells, further implying that ISL brought limited toxicity effects on HUVECs at low doses. (All values represented as mean ± SD, n = 6, *P<0.05, ** *P*<0.01 *versus* untreated control).

Since endothelial cells’ participation is a major factor contributing to angiogenesis, we examined whether ISL can inhibit the activation of endothelial cells induced by VEGF. As shown in Figure 1A, the proliferation of HUVEC was significantly increased in response to VEGF stimulation in the absence of ISL, while it was markedly suppressed after ISL administration. In contrast to endothelial cells, much higher concentrations of ISL were required to inhibit the proliferation of breast cancer cell line MCF-7 and MDA-MB-231(Figure 1B), suggesting that ISL had greater specificity as an inhibitor for VEGF-induced endothelial cell proliferation. To clarify whether the observed reduction in cell number of HUVECs resulted from the suppressed cell growth, we studied the effects of ISL on DNA synthesis by measuring Brdu incorporation. Our data indicated that ISL could significantly inhibit the DNA synthesis of HUVECs (Figure 1C). Meanwhile, cell cycle analysis revealed that ISL induced a G2/M arrest in HUVECs (Figure 1D). To determine whether ISL had cytotoxicity effect on HUVECs, we conducted the LDH cytotoxicity assay and cell morphology observation. The results revealed that ISL had little cytotoxicity effects on HUVECs from 5 to 20 µM (Figure 1E) and there has little morphological changes of HUVECs after ISL administration (Figure 1F).

### ISL Inhibits *in vitro* Angiogenesis

**Figure 2:**
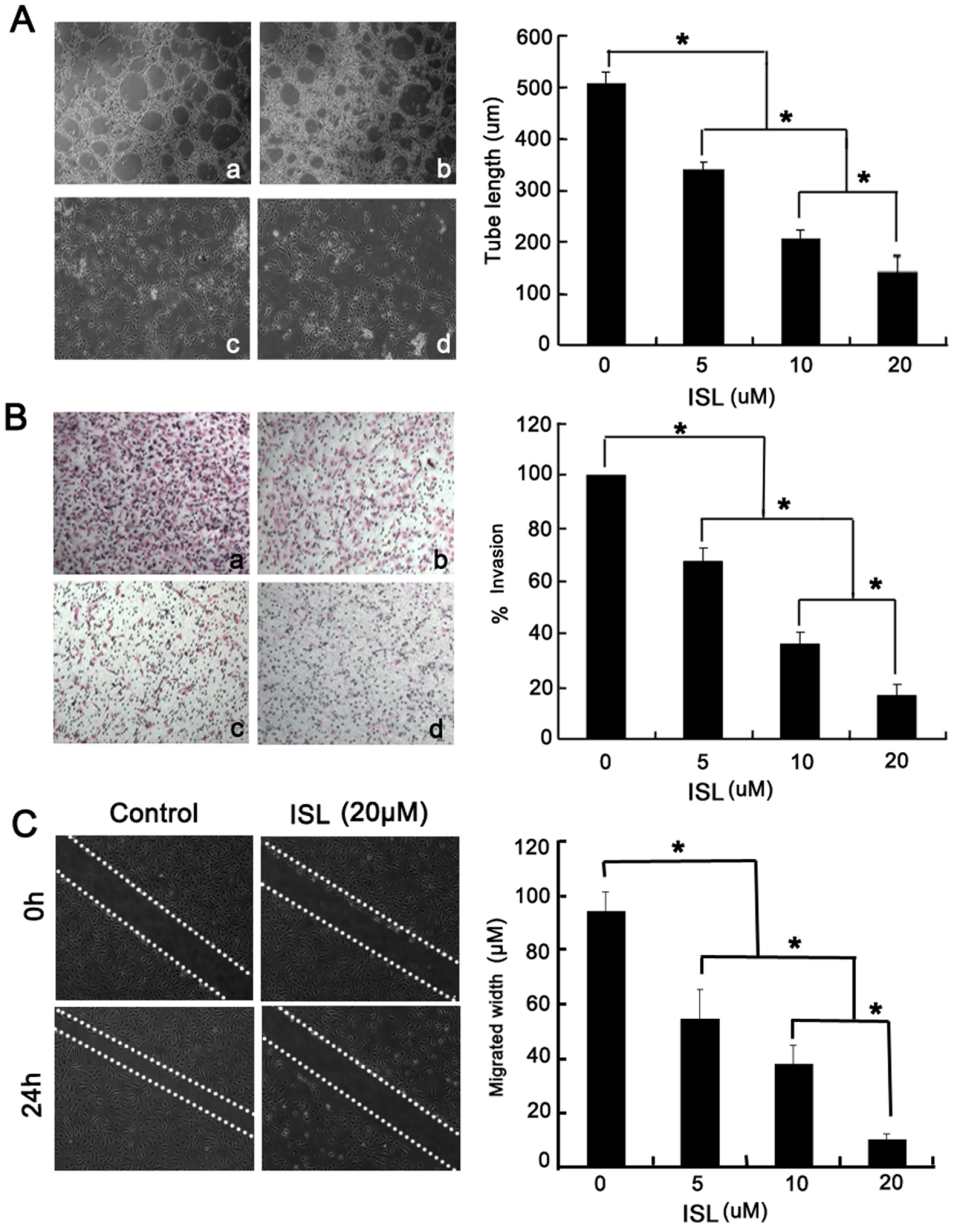
ISL inhibited VEGF-induced tube formation, invasion and migration. (A) HUVECs were seeded at a density of 1×10^4^ cells/well per 24-well plate. Plates were previously coated with Matrigel and stimulated with VEGF (20 ng/ml) in the presence or absence of ISL (a: 0; b: 5 µM, c: 10 µM; d: 20 µM) for 12 h. The results showed that ISL significantly abrogated the formation of capillary network; (B) HUVECs at a density of 5×10^5^ cells/ml were plated onto the upper membrane of 6-well transwell coated with Matrigel. After 24 h, cells that invasive to the opposite side of the membrane were counted (a: 0; b: 5 µM, c: 10 µM; d: 20 µM). The results revealed that ISL decreased invasive ability of HUVECs in a dose-dependent manner; (C) HUVECs at a density of 5×10^5^ cells/ml were seeded in a 6-well plate for wound healing assay. The results showed that ISL (20 µM) significantly inhibited endothelial cells migration under the stimulation of VEGF (All values represented as mean ± SD, n = 3, *P<0.05 *versus* untreated control).

Differentiation of endothelial cells into a tube-like structure is an important step for the formation of functional vessels. We investigated the effect of ISL on the morphological differentiation of endothelial cells into capillary-like structures using *in vitro* tube formation assay stimulated by VEGF. As shown in Figure 2A, a robust and complete tube network was observed in the VEGF alone group. However, ISL administration abrogated the width and the length of endothelial tubular structures in a dose-dependent manner.

Invasion of endothelial cells is also the key step required for neo-angiogenesis. VEGF is a potent stimulator for endothelial cells invasion and thus we used it as a chemo-attractant in the control group and drug-treated groups. When VEGF alone was presented in the lower chamber of a transwell plate, the endothelial cells could efficiently migrate through the micropores to the bottom of the membrane. However, when ISL was administrated on the top chamber, the migrated ratio of endothelial cells was significantly suppressed in a dose-dependent manner (Figure 2B). In addition, wound healing assay also revealed that the migration of endothelial cells was also inhibited after ISL administration for 24 h (Figure 2C). Overall, these results indicate that ISL has inhibitory roles in blocking migration and differentiation of endothelial cells induced by VEGF.

### ISL Suppresses Angiogenesis in Chick Aortic Ring Model

**Figure 3:**
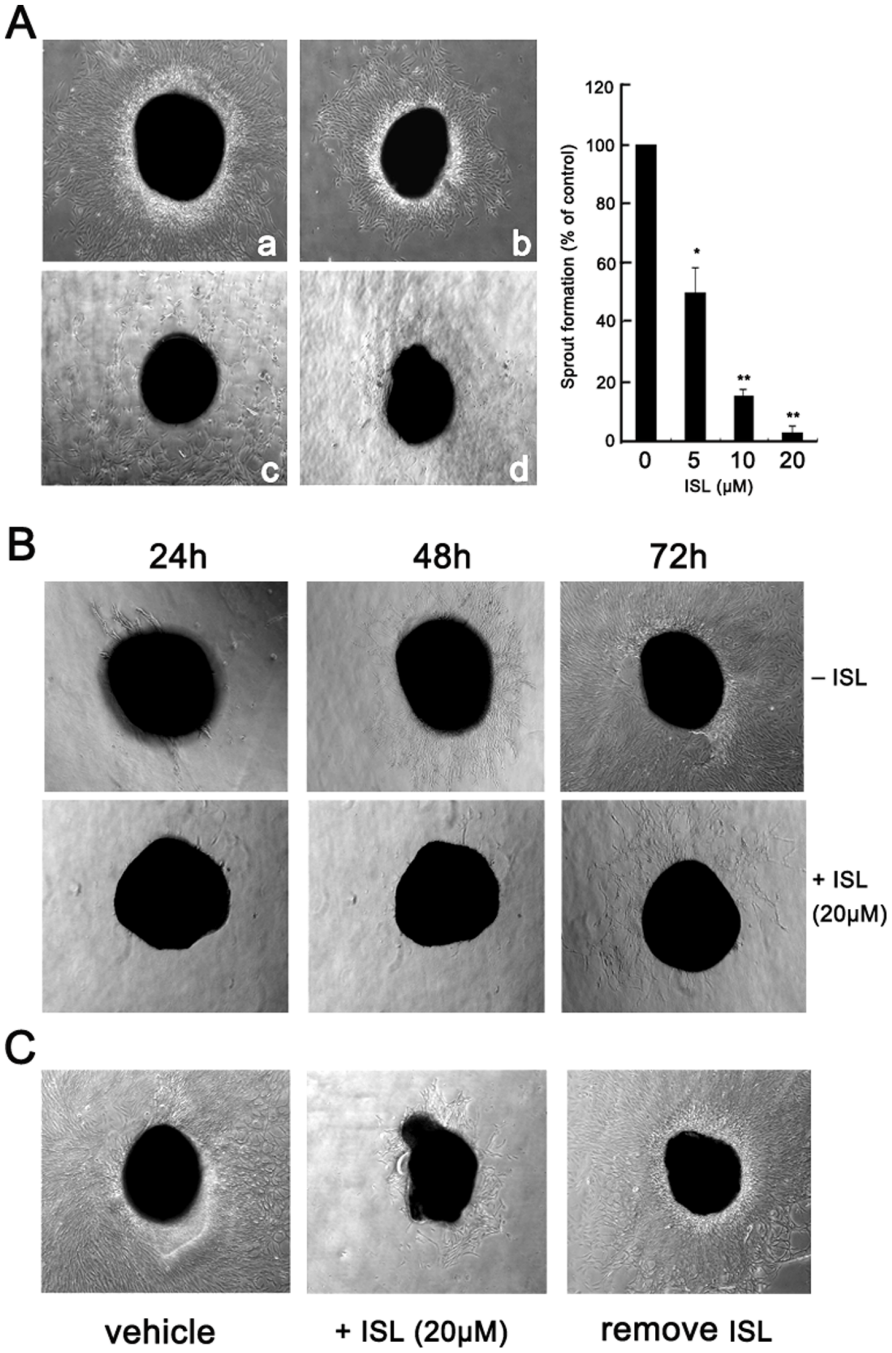
ISL suppressed sprout formation on the chick aortic ring. The chick aortic ring was embedded in Matrigel and fed with M199 serum free medium containing various concentrations of ISL in the presence of VEGF. (A) ISL exhibited dose-dependently inhibition effects (a: 0; b: 5 µM, c: 10 µM; d: 20 µM) on the sprout formation after 3 days; (B) ISL at 20 µM significantly inhibited sprout formation in a time-dependent manner from 24 h to 72 h; (C) Sprout inhibition effects of ISL was reversed after ISL removed from the culture system. Aortic rings were firstly incubated with ISL (20 uM ) for 48 h. The culture medium was then replaced with fresh medium without ISL and further incubation with VEGF stimulation for 72 h, the sprout was re-organized and growing, indicating ISL brought little toxicity effects on the normal tissues. (All values represented as means ± SD, n = 3, * *P*<0.05 *versus* untreated control).

To mimic the *in vivo* angiogenesis situation, the organotypic assay of chick aortic ring model was built to further confirm the potential angiogenesis inhibition effects of ISL. As shown in Figure 3A & B, ISL administration resulted in a dose and time dependent decrease in capillary sprout formation. The growing sprouts around the ring were shorter and cells migrated into the matrix were fewer in the 20 µM group, indicating that ISL might efficiently block neo-vascularization *in vivo*. In order to confirm whether the suppressed angiogenesis on chick aortic ring was due to the cytotoxic or proliferation inhibition effects of the drug, ISL was withdrawn after its exposure to aortic rings. As shown in Figure 3C, the re-generation of the sprout around the ring further indicated that ISL brought little toxicity effects on normal tissue or cells.

### ISL Blocks VEGF Expression *via* Promoting HIF-1α Proteasome Degradation

**Figure 4:**
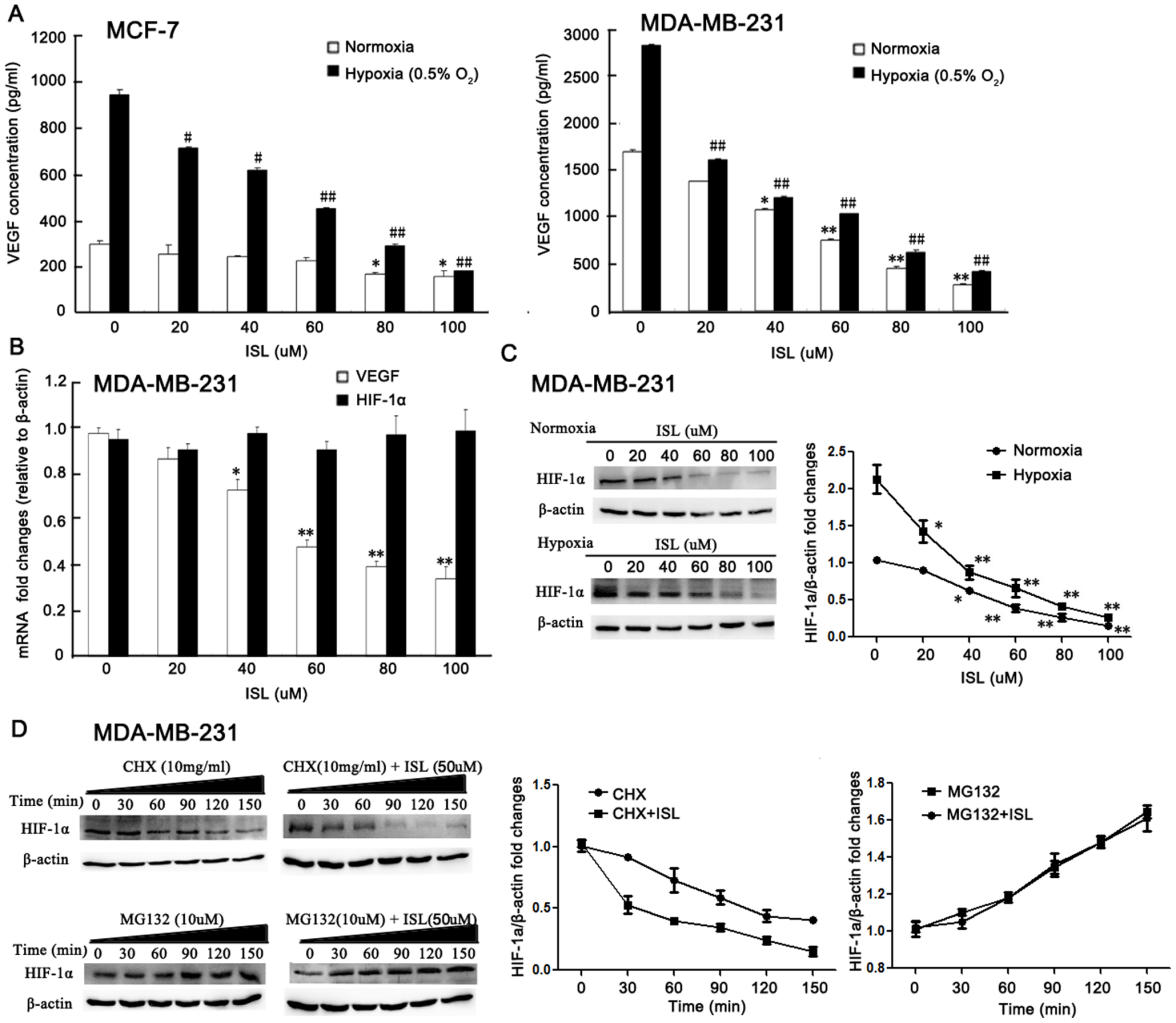
ISL blocked VEGF expression of breast cancer cells via promoting HIF-1α proteasome degradation pathway. (A) The VEGF concentration in the supernatants of breast cancer cells after ISL treatment was detected by Elisa method. The results revealed that ISL could significantly inhibit VEGF secretion in a dose-dependent manner under both normoxia and hypoxia condition; (B) RT-PCR assay showed that ISL could inhibit VEGF mRNA transcription in a dose-dependent manner, however, had little effects on HIF-1α mRNA level; (C) Western blotting results showed that ISL could significantly inhibit HIF-1α protein expression under both normoxia and hypoxia condition; (D) ISL inhibited HIF-1α protein expression *via* promoting its proteasome degradation pathway. Cychloheximide (CHX) was firslty administrated on breast cancer cells to inhibit protein synthesis. The expression of HIF-1α in both control and ISL treated cancer cells were validated by western blotting. The results showed that the HIF-1α degradation speed was much faster in ISL treated groups than in control groups. Meanwhile, MG132 was administrated on breast cancer cells to block the proteasome activity. The results indicated that the accumulation quantities of HIF-1α were comparable in both control and ISL treated groups, indicating that the proteasome degradation was mainly accounting for HIF-1α and VEGF down-regulation induced by ISL. (All values represented as means ± SD, n = 3, *,^#^ *P*<0.05, **,^##^ *P*<0.01 *versus* untreated control).

VEGF has been identified as the most important pro-angiogenic factor during cancer growth. In order to see whether ISL would inhibit VEGF secretion in breast cancer cells, we detected VEGF concentration in cell supernatants after ISL administration. The results showed that under both normoxia and hypoxia condition, ISL could significantly inhibit VEGF secretion from breast cancer cells MCF-7 and MDA-MB-231(Figure 4A). To determine whether the decreased secretion was caused by down-regulated VEGF expression, we detected VEGF mRNA level by RT-PCR method. The results revealed that ISL could greatly suppress VEGF mRNA level, but had little effects on its upstream gene HIF-1α expression (Figure 4B). As HIF-1α was reported to be the most important gene controlling VEGF expression, we therefore intend to see whether ISL could also inhibit HIF-1α expression. Western blotting results indicated that ISL could inhibit HIF-1α expression under both normoxia and hypoxia condition (Figure 4C). Besides transcription regulation, proteasome degradation was also one of important regulator of intracellular HIF-1α protein level. In order to see whether the decreased HIF-1α protein level was caused by accelerated proteasome degradation by ISL, we firstly added cycloheximide, a protein synthesis inhibitor, to treat breast cancer cells with or without ISL. The results showed that in MDA-MB-231 breast cancer cells, the HIF-1α degradation speed was much faster in the ISL treated group than that in the control group, indicating that the proteasome degradation pathway might be activated. Meanwhile, the proteasome inhibitor MG132 was then administrated to MDA-MB-231 cells with or without ISL. The results showed that the HIF-1α accumulation level was comparable in ISL treated or untreated groups, indicating that the accelerated proteasome degradation pathway was mainly accounting for HIF-1α and VEGF down-regulation induced by ISL (Figure 4D).

### ISL Inhibits the VEGFR-2 Signaling Pathway in Endothelial Cells

**Figure 5:**
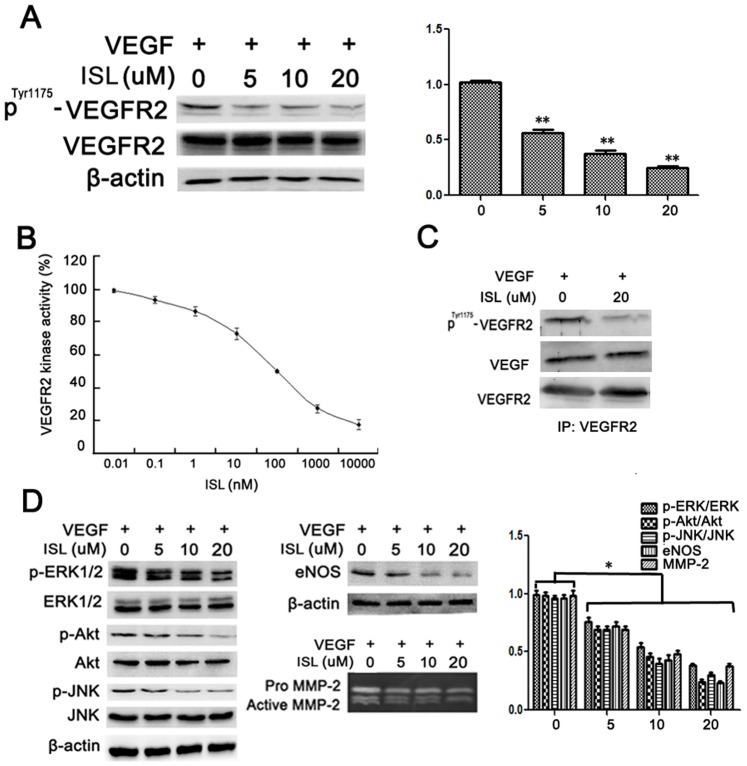
ISL suppressed VEGFR-2 kinase activity and its downstream signaling. (A) HUVECs were treated with various concentrations of ISL for 12 h under the stimulation of VEGF (20 ng/ml). Western blotting results showed that the p-VEGFR-2 expression was gradually down-regulated with the increasing dose of ISL; (B) ISL inhibited VEGFR-2 kinase activity. VEGFR-2 and various concentrations of ISL were incubated in kinase reaction buffer in 96-well plates coated with a poly-Glu-Tyr substrate. Phosphorylation of the substrate was monitored with a purified phosphotyrosine specific monocolonal antibody conjugated to horseradish peroxidase followed by chromogenic reaction with horseradish peroxidase substrate. The inhibition IC 50 of ISL on VEGFR-2 activation was determined about 100 nM; (C) Whole-cell extracts were collected and analyzed by Co-IP assay and Western blotting using antibodies against VEGF, VEGFR-2 and P^Tyr1175^-VEGFR-2. The results showed that ISL did not interfere with VEGF binding to VEGFR-2; (D) The VEGFR-2 downstream signalings including p-ERK/ERK, p-JNK/JNK, p-AKT/AKT and eNOS were also inhibited after ISL administration demonstrated by Western blotting. Meanwhile, the MMPs activities in the supernatants of HUVECs treated with ISL were also analyzed by gelatin zymography. The results indicated that the MMP-2 activity was also down-regulated by ISL. (All values represented as mean ± SD, n = 3, *P<0.05, ** *P*<0.01 *versus* untreated control).

Since ISL inhibited VEGF-induced proliferation, migration and tube formation of endothelial cells, we next investigated whether ISL blocks the VEGF-induced signaling cascade pathways in endothelial cells. Strong evidence demonstrated that VEGFR-2 tyrosine phosphorylation is a key step in maintaining endothelial cells’ normal functions and formation of blood vessels. Thus we tested the effects of ISL on VEGF-induced tyrosine phosphorylation of VEGFR-2. As shown in Figure 5A, the level of tyrosine phosphorylated VEGFR-2 was significantly inhibited by ISL in a dose dependant manner in comparison with cells only administrated with VEGF. To determine whether the inhibition of tyrosine phosphorylation of VEGFR-2 by ISL is induced by the inhibition of the kinase activity of VEGFR, we performed the *in vitro* tyrosine kinase assay based on the ELISA method. The results showed that ISL significantly inhibited the VEGFR-2 kinase activity, with an IC50 of 100 nM (Figure 5B), implying that ISL could directly interact with VEGFR-2 *via* competing with ATP binding. In order to investigate whether ISL would bring influence to VEGF binding activity with VEGFR-2, we carried out Co-IP assay. The results showed that after ISL treatment, the level of VEGF binding to VEGFR-2 had little changes, indicating that the suppressed VEGFR-2 activity was not attributed to decreased VEGF binding (Figure 5C). Meanwhile, multiple downstream signaling of VEGFR-2 including phosphorylation of ERK1/2, JNK, AKT, eNOS and MMP-2 were also inhibited, which further revealed that the ISL might inhibit HUVECs proliferation *via* directly interfering with VEGFR-2 (Figure 5D).

### ISL Inhibits Breast Cancer Neo-angiogenesis *in vivo*

**Figure 6:**
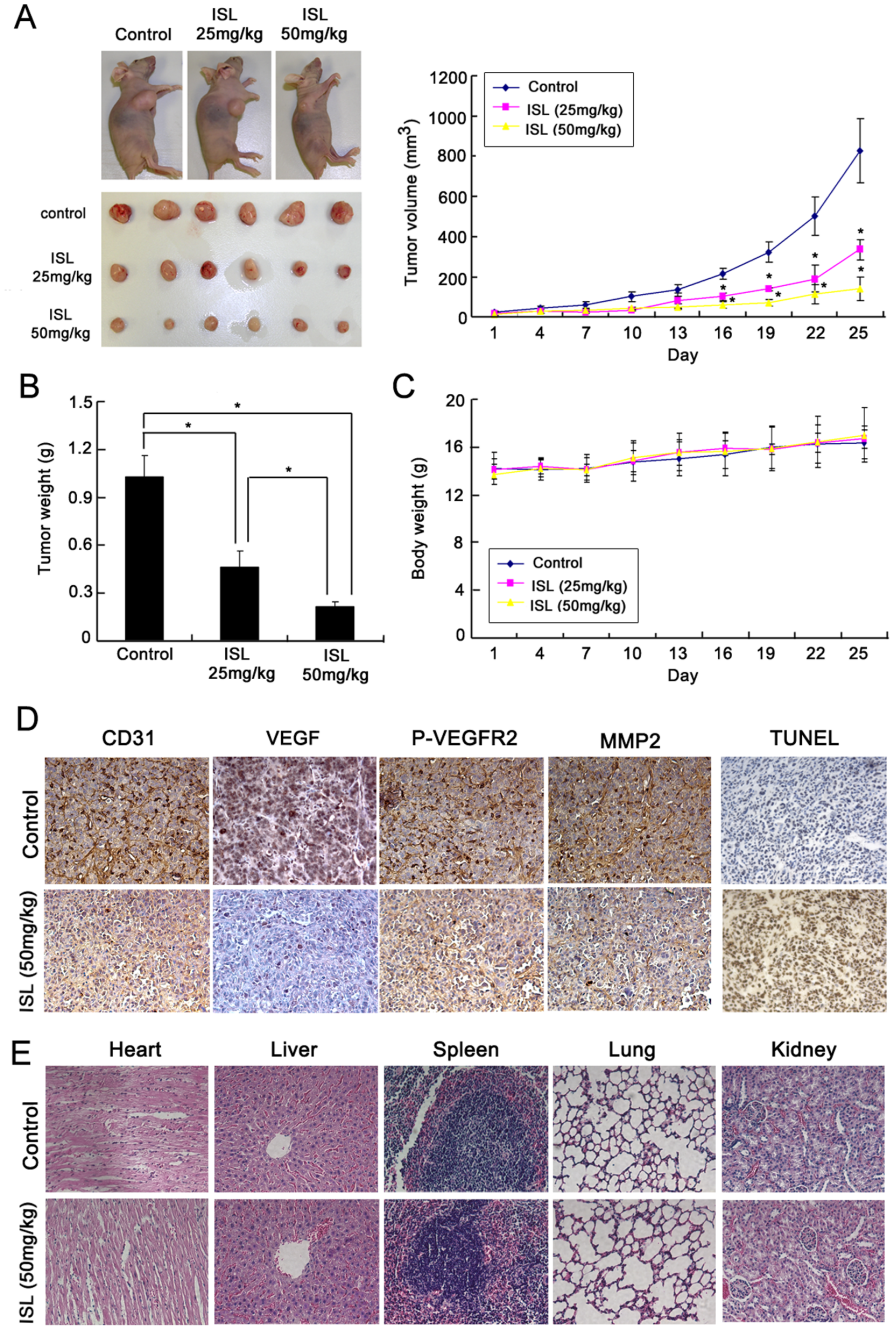
ISL inhibited tumor growth and angiogenesis on MDA-MB-231 breast cancer xenografts. (A) Nude mice bearing breast cancer were treated with the vehicle or ISL (25 and 50 mg/kg/d). The results showed that ISL significantly attenuated breast cancer growth in a dose-dependent manner; (B) The tumor weights in ISL- treated group were significantly decreased in comparison with the vehicle control; (C) The body weights between control and ISL-treated group had little differences, indicating ISL might have little toxicity effects on mice; (D) The tumor tissues removed from mice were processed for immunohistochemistry detected with antibodies for CD31, VEGF, p-VEGFR-2 and MMP-2. The results showed that the tumor MVD was significantly inhibited by ISL. Meanwhile, ISL significantly suppressed the expression of VEGF, p-VEGFR-2 and MMP-2 *in vivo*; (E) HE analysis demonstrated that ISL had little influences on the micro-morphology of normal tissues including heart, liver, spleen, lung and kidney (All values represented as means ± SD, n = 6, * *P*<0.05, *versus* control).

To determine whether ISL has anti-angiogenesis activity *in vivo*, breast cancer xenografts were built by seeding MDA-MB-231 cancer cells into the mammary glands of nude mice. ISL was given to mice by intraperitoneal injection at 25 mg/kg/d and 50 mg/kg/d. Vehicle treated control mice showed a rapid increase in tumor growth. However, ISL low and high dose administration significantly suppressed tumor growth from day 16 after treatment, with a 50–65% inhibition ratio compared to the vehicle groups (Figure 6A). The tumor weights in ISL groups were also significantly reduced in comparison with control group (Figure 6B). However, no significant body weight loss was observed, indicating that ISL might have little toxicity effects by *in vivo* application (Figure 6C). To determine whether ISL could inhibit angiogenesis and VEGFR-2 expression *in vivo* as what we observed in the *in vitro* experiments, we detected tumor microvessel density (MVD), VEGF, p-VEGFR-2, MMP2 expression and apoptosis by immunohistochemistry method. The results showed that the MVD was significantly decreased in the ISL treated tumor sample when compared to the vehicle control group, suggesting that the tumor growth was partly due to the blood vessel suppression caused by ISL. Meanwhile, the VEGF, pVEGFR-2 and MMP2 expression were also significantly down-regulated in ISL treated tumor samples. In addition, TUNEL analysis revealed that in ISL treatment group, there has a higher apoptosis ratio in comparison with the control group (Figure 6D). In order to validate whether ISL administration resulted in toxicity on normal tissues, the heart, liver, spleen, lung and kidney tissues were collected for morphological detection by HE method. The results showed that little significant morphological changes on these tissues were observed, which were consistent with our *in vitro* study (Figure 6E). Taken together, these results suggest that ISL can inhibit tumor progression and MVD through suppression of the VEGFR-2 signaling pathway.

### ISL Locates within the ATP-binding Sites of VEGFR-2

**Figure 7:**
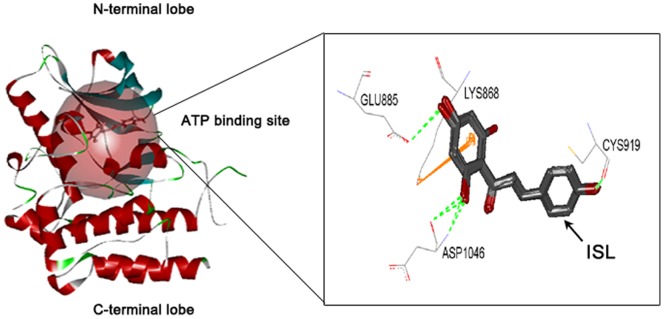
ISL interacted with the ATP-binding site of VEGFR-2 kinase domain. The CDOCKER module in Discovery studio (DS) 2.1 was applied to predict the binding mode of ISL with the ATP-binding domain of VEGFR-2. The results revealed that ISL could stably bind to the ATP-binding pocket near the hinge region. Detailed interaction mode were displayed in the upper panel, where ISL could form 2–3 potential hydrogen bonds with the residues Glu885 and Asp1046. A **π-π** stacking interaction with Lys868 is also occurred. The other benzene ring of ISL could form potential hydrogen bonds with Cys919, which benefits the balance of ISL in VEGFR-2 binding.

We next analyzed the binding pattern between ISL and VEGFR-2 to further understand how ISL inhibits the activation of VEGFR-2 kinase and its downstream signaling pathways. The ATP-binding site within VEGFR-2 was defined as the docking site. The RMSD of internal ligand (Compound 20d) re-docking into the ATP binding site of VEGFR-2 (3VHE) with CDOCKER algorithm was 0.38, implying that the algorithm was suitable for docking analysis. The docking results showed that ISL presenting a unique binding mode to the ATP binding site of VEGFR-2 with strong stability (RMSD = 0.0326∼1.5699 (ten random poses)) and strong binding activity (CDOCK_ENERGY = 33.541) compared to the internal ligand (CDOCK_ENERGY = 44.737). As shown in the Figure 7, ISL displayed unique binding features in the ATP binding domain. On one side of ISL, 2–3 potential hydrogen bonds are formed with the residues Glu885 and Asp1046 of VEGFR-2, accompanying with a **π-π** stacking interaction with Lys868. Meanwhile, on the other benzene ring of ISL, it could form hydrogen bonds with Cys919, which contributes to the stability and balance of ISL binding to the protein. All these results suggested that ISL might be a potent VEGFR-2 inhibitor.

## Discussion

Angiogenesis plays an important role in cancer development and metastasis. Therefore angiogenesis inhibition has become a promising strategy for malignancy treatment in addition to conventional therapies such as chemotherapy and radiotherapy. However, current anti-angiogenic agents have relative hazardous effects in chronic applications. Finding less toxic phytochemicals targeting tumor angiogenesis has become an important direction in cancer research. ISL is a charlcone-type dietary flavonoid. The anti-tumor activity of ISL has recently been reported in various types of cancer including breast, prostate, colon, oral, cervical and leukemia [16]–[19], [27]. The discovered anti-cancer mechanisms of ISL include cell proliferation inhibition, cell cycle arrest, inflammation suppression, apoptosis induction and elevation of oxidative stress. Several studies also reported that ISL could inhibit neo-angiogenesis [23], [25]. However, whether or not ISL can inhibit cancer neo-angiogenesis and its underlying molecular mechanisms are still remained largely unknown.

At the cellular level, our results showed that ISL could inhibit various steps of angiogenesis, including VEGF-induced endothelial cell proliferation, tube formation, migration and aortic ring sprout formation. The inhibitory activity of ISL on endothelial cell functions is not likely due to the cytotoxicity effects. This was reflected by our observation that ISL has a rather specific activity toward actively proliferating HUVECs in comparison to non-endothelial cells and ISL brought little LDH release from HUVECs. To better understand the mechanism by which ISL inhibit cancer growth and angiogenesis, here we investigated the influences of ISL on the most critical signaling pathway VEGF/VEGFR-2 that stimulate angiogenesis. Our results found that ISL could inhibit VEGF expression in both MCF-7 and MDA-MB-231 breast cancer cell lines, especially under hypoxia condition. The primary regulator of VEGF expression in response to hypoxia is HIF-1α. HIF-1α activates VEGF gene expression by binding to the hypoxia response element in the VEGF promoter region. The level of HIF-1α expression is mainly determined by the rate of protein synthesis compared to protein degradation, which was mediated by poly-ubiquitination and subsequent digestion by proteasome. Herein, we demonstrated that ISL could inhibit HIF-1α expression under both normoxia and hypoxia conditions, while there had little changes on its mRNA expression level, indicating that the proteasome degradation pathway might be activated. This possibility was supported by the comparable level of HIF-1α regardless of ISL treatment in the presence of proteasome degradation inhibitor MG132. On the other hand, it was observed that HIF-1α degradation was accelerated under the treatment of both ISL and protein synthesis inhibitor CHX, indicating that the HIF-1α proteasome degradation pathway is the main mechanism accounting for the decreased VEGF expression induced by ISL.

VEGFR-2 has been reported to be the critical molecule responsible for maintaining endothelial normal functions including proliferation, migration, differentiation, capillary network formation and vascular permeability [28]. Upon ligand binding with VEGF, VEGFR-2 undergoes autophosphorylation and becomes activated. Meanwhile, VEGFR-2 activation can also trigger intracellular signaling by phosphorylation with other proteins such as Akt, Jnk and Erk, which were reported to be elevated in tumors and correlated with tumor progression [29], [30]. A growing list of inhibitors of angiogenesis targeting at VEGFR-2 have been designed. However, little is known about whether natural products can inhibit the VEGFR-2 kinase activity. In our study, treatment with ISL markedly reduced the phosphorylation form of VEGFR-2. Meanwhile, the VEGFR-2 tyrosine kinase activity was also significantly inhibited by ISL. Although the inhibitory efficacy of ISL is much lower than that of sunitinib (10 nM) [31], it might be more suitable for long-term application as a dietary compound. These findings suggest that the anti-angiogenic effect of ISL is mediated, at least in part, by the reduction of VEGFR-2 activity. In order to investigate whether the decreased VEGFR-2 activity is caused by reduced binding of VEGF, we conducted Co-IP assay. The results found that ISL brought little influences on the level of VEGF binding to VEGFR-2, indicating that the decreased VEGFR-2 activity might be due to the direct inhibition effects of ISL.

As the downstream activators of VEGFR-2, the P42/44 ERK, JNK-MAPK and Akt pathways are considered as the most critical pathways for maintaining endothelial cells survival. In particular, P42/P44 ERK activation is considered as an absolute requirement for VEGF-induced angiogenesis. Blocking ERK activation resulted in significant proliferation inhibition effects and apoptosis on endothelial cells [30]. JNK activation also results in cell proliferation by promoting nuclear activation of c-Jun [32]. Akt is a serine/threonine protein kinase that plays a key role in multiple cellular processes such as cell proliferation, cell cycle, apoptosis and cell migration [33]. Our results showed that ISL administration inhibited all these three pathways, indicating that they might cooperatively contribute to the proliferation inhibition effects induced by ISL.

Disruption of the basement membrane is also a key step in angiogenesis an metastasis. Matrix metalloproteinases (MMPs) play an important role in degrading the proteins in the extracellular matrix such as collagens and gelatins [34]. At least 17 MMP members have been identified in humans. They are classified into subgroups of collagenases (MMP-1, MMP-8 and MMP-13), gelatinases (MMP-2, MMP-9), stromelysins (MMP-3, MMP-7, MMP-10, MMP-12), membrane-type MMPs (MMP-14, MMP-15, MMP-16, MMP-17) and others (MMP-11, MMP-19, MMP-20) [35]–[37]. Among them, MMP-2 has been shown to be associated with angiogenesis during cancer development and metastasis [38], [39]. Our results also demonstrated that ISL could significantly suppress MMP-2 expression stimulated by VEGF in HUVECs. Since MAPK pathways were reported correlating to the activation and expression of MMPs [40], the MMPs inhibition effects of ISL on HUVECs might be also attributed to the suppressed activity of P42/44 ERK and JNK pathways.

**Figure 8:**
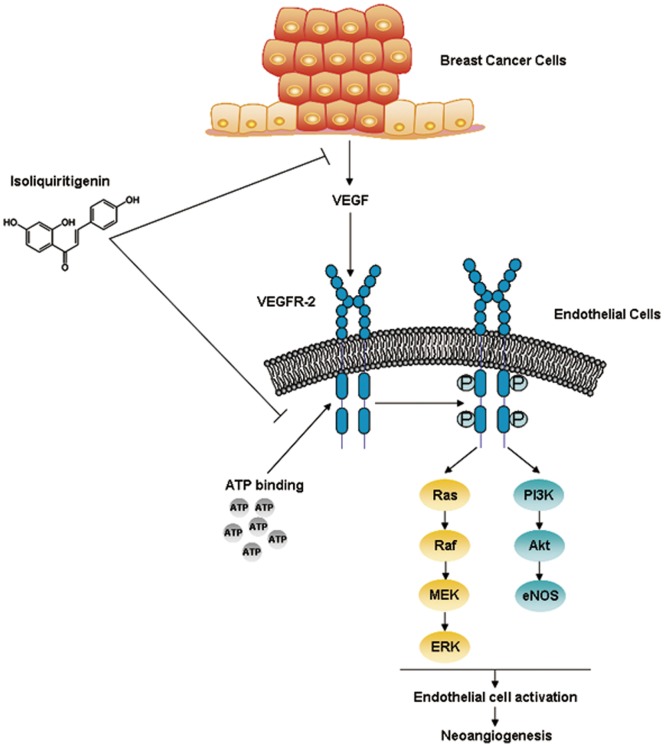
Overall scheme of the regulatory network involved in the inhibitory effect of ISL on breast cancer neoangiogenesis.

To thoroughly understand how ISL interacted with VEGFR-2 to inhibit cancer angiogenesis, we further examined the structure-based interaction between ISL and VEGFR-2 by *in silico* analysis. Structurally, VEGFR-2 consists of 1356 amino acids in humans, and can be separated into the N-terminal lobe and the larger C-terminal lobe [41]. To be specific, the N-terminal side at residues 820–920 is composed of a twisted β sheet and one α helix (αC). Among the five anti-parallel strands (β1–β5) of β sheet, three (β1–β3) strands are curved and curl over the other two (β4–β5). The C-terminal side at residues 921–1168 contains two anti-parallel β strands (β7–β8) as well as seven α helices (αD, αE, αE-F, αF, αG, αH, αI). The β7 and β8 are located at the top of the C-terminal side bordering β structure of the N-terminal [42]. In addition, the architecture of VEGFR-2 involves several important loop domains including glycine-rich loop (also refers to nucleotide binding loop) at residues 841–846, the catalytic loop at residues 1026–1033, and the activation loop at residues 1046–1075 [42], [43]. Notably, the ATP-binding domain lies between N-terminal lobe and C-terminal lobe of the kinase fold. This site and less conserved surrounding sites are especially important for anti-VEGFR2 agent design [44]. Many approved VEGFR-2 inhibitors, such as sorafenib and sunitinib, exert their inhibitory effects through purely or partially competing with ATP to bind with the ATP pocket within the catalytic domain and subsequently suppressing the receptor autophosphorylation. Particularly, through docking analysis of present VEGFR-2 inhibitors, the active sites around the ATP-binding domain of VEGFR-2 are hypothesized consisting of three hydrophobic regions (Region 1–3) as well as one polar region (region 4). The first hydrophobic pocket Region 1 contains residues including Val846, Ala864, Val897, Val914, Phe916 and Leu1033, adjacent to the hinge region. Larger Region 2 is composed of residues such as Leu887, Val896, Val897, Leu1017 and Phe1047. Between the Region 1 and the Region 2, residues Lys866, Glu883 as well as Asp1044 are also critical for receptor activation. Region 3 contains only a few residues including Leu838 and Phe916. The unique polar region involves several residues such as Asn921, Cys1043, Arg1030 and Asn1031 [45], [46]. Our docking results showed that ISL could bind to the residues of Glu 885, Lys 868, Asp 1046 and Cys 919 with great stability and binding energy. These involved residues are very close to the above stated active sites, implying that ISL could inhibit VEGFR-2 activity by directly interacting with the ATP-binding site of VEGFR-2. The inhibitory action of ISL on cancer angiogenesis was summarized in Figure 8.

To evaluate the effect of ISL on *in vivo* tumor angiogenesis, we built breast cancer xenografts on nude mice. The results demonstrated that ISL could significantly inhibit breast cancer growth, accompanied with a reduced MVD on tumor samples. Based on the *in vitro* results, the reduced MVD may be due to the inhibited VEGFR-2 activation and its induced suppression effects on both endothelial cells and other cell types such as macrophages, leukocytes and tumor cells, which play an important role in neo-angiogenesis during tumor development. Our results showed that both P-VEGFR-2 and its downstream signaling molecules expression were significantly down-regulated in ISL treatment tumor tissues, which were consistent with our *in vitro* findings. Meanwhile, the apoptosis ratio in ISL-treated samples was elevated, which might be due to the insufficient blood nutrients. In addition, little significant morphological changes on normal tissues were found out, implying that ISL is safe when consumed *in vivo*.

In recent years, a great emphasis has been focused on the development of dietary botanicals that can be consumed in daily life as chemopreventive or chemotherapeutic agents. Flavonoids, as common compounds widely found in fruits, vegetables, nuts, seeds and flowers, have been reported possessing substantial anti-carcinogenic and anti-mutagenic activities due to their antioxidant and anti-inflammatory properties [47], [48]. However, little is known about the key molecular targets underlying their chemopreventive or therapeutic activities against various cancers. Our findings indicated that the dietary compound ISL might be considered as a potent VEGFR-2 inhibitor and be chronically used as supplementary agents for angiogenesis inhibition in breast cancer therapy. However, further study is needed to evaluate the prevention role of ISL on inhibiting angiogenesis by a carcinogen-induced or genetically engineered tumor model. The interaction between ISL and cancer conventional therapies also requires deep further study in the future.
