# Expression of Concern: Dietary Compound Isoliquiritigenin Inhibits Breast Cancer Neoangiogenesis *via* VEGF/VEGFR-2 Signaling Pathway

**DOI:** 10.1371/journal.pone.0343779

**Published:** 2026-02-26

**Authors:** The PLOS One Editors

After publication of this article [1], concerns were raised about Fig 6, animal welfare, the reported statistical analysis, and the peer review for this article.

In Fig 6D, the CD31 Control panel partially overlaps with the P-VEGFR2 Control panel. First author ZW stated that an error was made in the preparation of Fig 6D and provided a revised version of Fig 6 presenting the correct image for the P-VEGFR2 Control panel from the original study, along with the underlying images for Fig 6 in S1 File.

The methodology section of [1] does not report on animal handling, humane endpoints, or the ethical approval obtained for this study. First author ZW stated that throughout the entire experimental period, animal health and behavior were systematically monitored twice per week by trained laboratory technicians. The parameters monitored included body weight, food intake, and normal behavior. If the mice were showing any symptoms of dyspnea, tremor, convulsion, paralysis or coma, had tumors with the volume exceeding 1,000 mm^3^, or lost more than 20% body weight, the mice were euthanized by intraperitoneal injection of an overdose of ketamine and xylazine. The approval number of the ethics approval document issued by the Committee on the Use of Live Animals in Teaching and Research of the University of Hong Kong is 2062−10. With the additional information provided by first author ZW PLOS considers this concern resolved.

Regarding the statistical concerns, the Materials and Methods section of [1] only reports the use of Student’s t-tests, but this approach is inappropriate for the analysis of data involving repeated measures, multiple groups, or small sample sizes. Furthermore, the article did not report whether and how the reported P values were corrected for multiple testing. First author ZW stated that the Data Analysis subsection within the Materials and Methods section is incomplete and provided the following update to the Data Analysis subsection:

Comparisons between two groups were performed using two-tailed Student’s t-tests. For three or more groups, one-way or two-way Analysis of Variance (ANOVA) was employed, followed by Tukey’s or Bonferroni’s post hoc test for multiple comparisons. Statistical significance was considered when the P value < 0.05. The tumor volume data presented in Fig 6A were analyzed using a two-way repeated-measures ANOVA. The data were first assessed for normality using the Shapiro-Wilk test. While minor deviations from normality were noted in a few instances, we proceeded with ANOVA given its well-established robustness to such slight departures, particularly with balanced group designs. Post-hoc comparisons were conducted using the Bonferroni correction. A one-way ANOVA was applied to analyze the tumor weight data in Fig 6B. Normality was confirmed across all groups via the Shapiro-Wilk test.

First author ZW provided individual-level underlying data for Figs 6A, 6B, and 6C (S2 File). After editorial assessment of the updated Data Analysis section and the provided individual-level underlying data, PLOS considers the concern about the statistical analysis resolved for Fig 6. In the absence of underlying data for the other figures, the reliability of the statistical analysis for the results presented in Figs 1-5 cannot be verified.

During the editorial reassessment of this article [1], PLOS identified concerns regarding the article’s peer review. The Editors have no evidence of any author involvement in the peer review concerns. PLOS regrets that this issue was not identified prior to the article’s publication.

With this notice, PLOS considers the concerns with Fig 6 and the animal welfare reporting resolved. The *PLOS One* Editors issue this Expression of Concern to inform readers of the outstanding concerns with the reliability of the statistical analysis of results presented in Figs 1-5 and the peer review of this article, and to advise readers to interpret the findings reported in [1] with caution.

Since the publication of this article [1], references 11 [2] and 39 [3] have been retracted [4,5].

**Fig 6:**
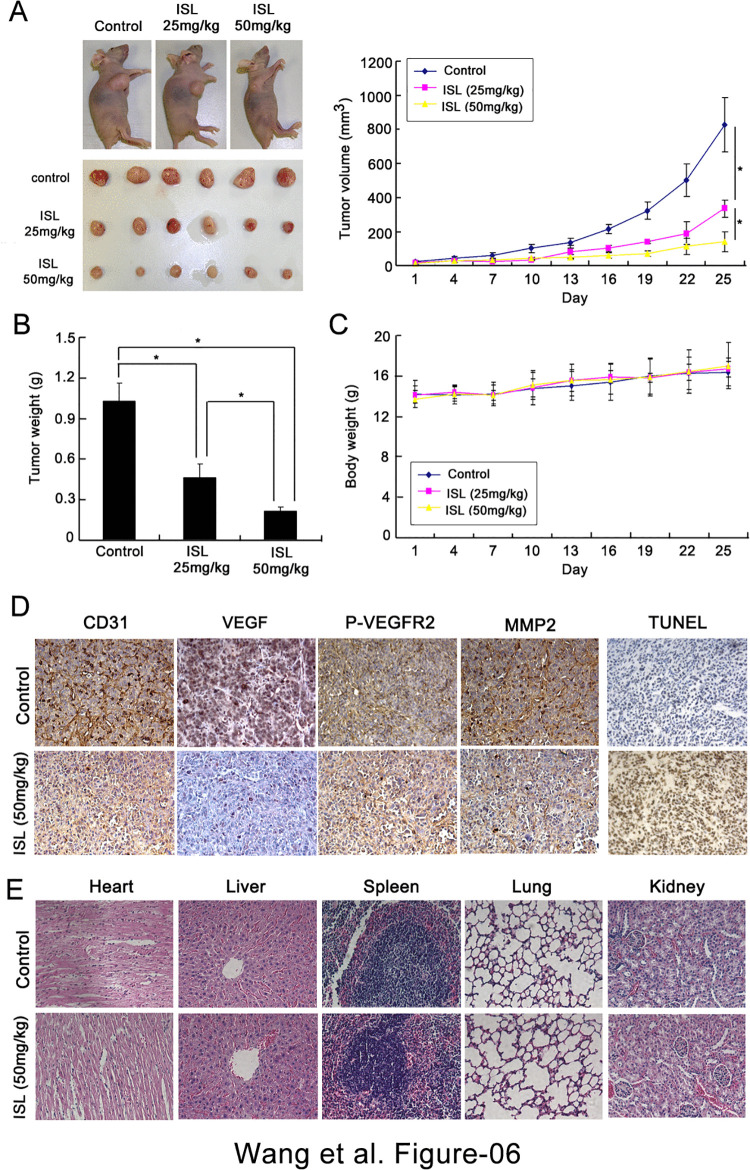
ISL inhibited tumor growth and angiogenesis on MDA-MB-231 breast cancer xenografts. **(A)** Nude mice bearing breast cancer were treated with the vehicle or ISL (25 and 50 mg/kg/d). The results showed that ISL significantly attenuated breast cancer growth in a dose-dependent manner; **(B)** The tumor weights in ISL- treated group were significantly decreased in comparison with the vehicle control; **(C)** The body weights between control and ISL-treated group had little differences, indicating ISL might have little toxicity effects on mice; **(D)** The tumor tissues removed from mice were processed for immunohistochemistry detected with antibodies for CD31, VEGF, p-VEGFR-2 and MMP-2. The results showed that the tumor MVD was significantly inhibited by ISL. Meanwhile, ISL significantly suppressed the expression of VEGF, p-VEGFR-2 and MMP-2 *in vivo*; **(E)** H&E analysis demonstrated that ISL had little influences on the micro-morphology of normal tissues including heart, liver, spleen, lung and kidney. All values represented as mean ± SD; n = 6; * *P* < 0.05, as determined by Analysis of Variance (ANOVA) followed by either Tukey’s or Bonferroni’s post hoc test for multiple comparisons.

## Supporting information

S1 File Original images for Figs 6D and 6E and images from two replicates. (ZIP)

S2 File Individual-level underlying data for Figs 6A, 6B, and 6C. (ZIP)
