# Supplementary material for: Expression of Concern: Dietary Compound Isoliquiritigenin Inhibits Breast Cancer Neoangiogenesis via VEGF/VEGFR-2 Signaling Pathway
Source: PLoS One. 2026 Feb 26;21(2):e0343779. doi: 10.1371/journal.pone.0343779 (PMC12944767; doi:10.1371/journal.pone.0343779)

Figure 6E-1st repeat (used in the Manuscript)

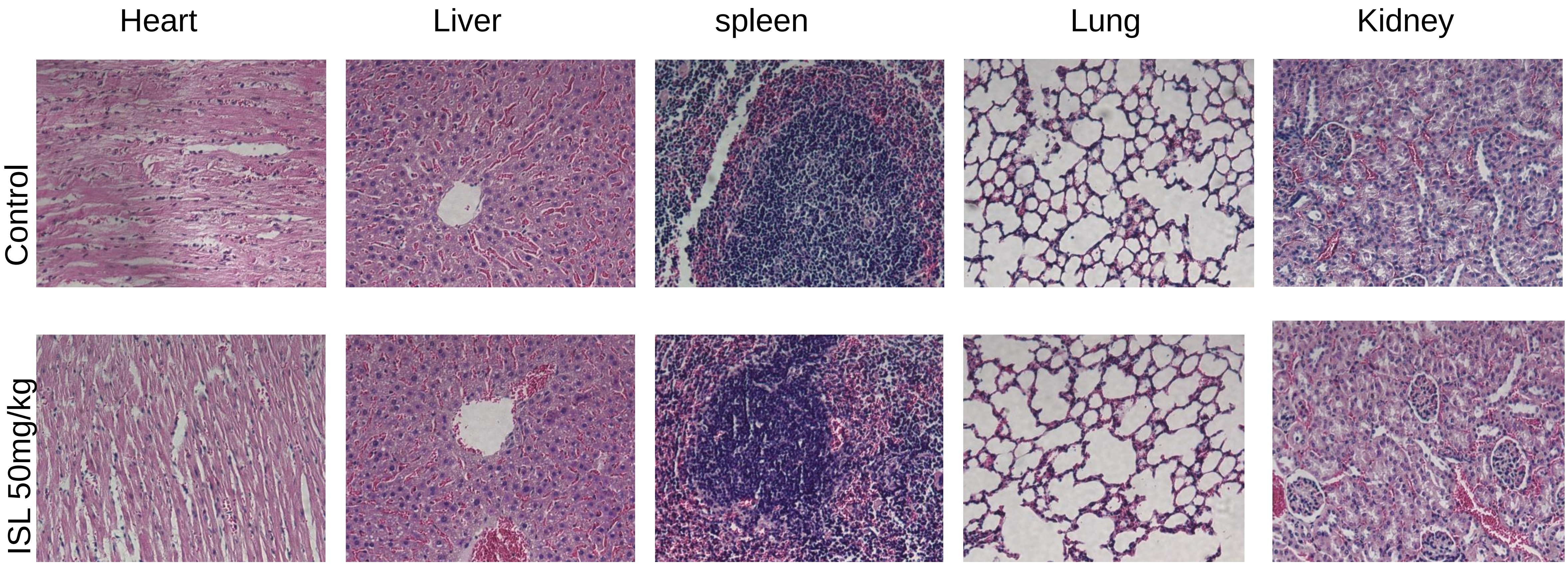

Figure 6E-2nd repeat (other replicates)

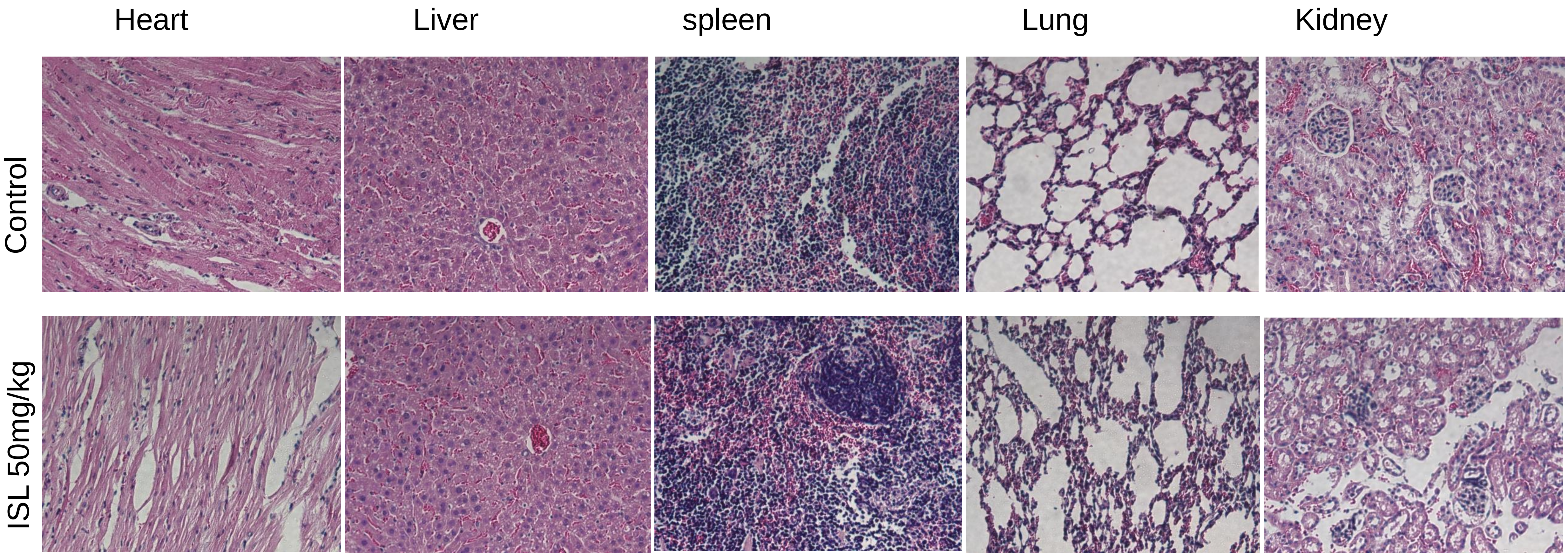

Figure 6E-3rd repeat (other replicates)

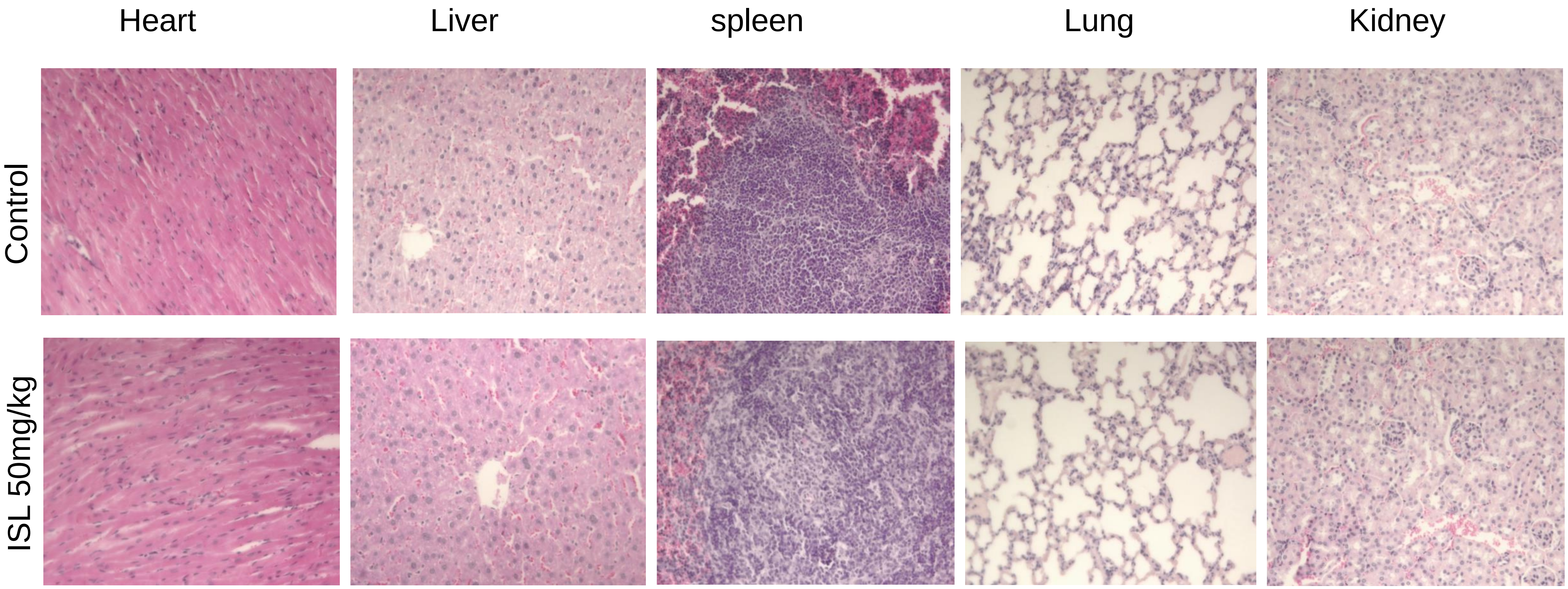

Supplement: S1 File — (ZIP) [file pone.0343779.s001.zip › S1 File/other replicates for Figure 6E.pdf]
