# Supplementary figures and images for: Expression of Concern: Dietary Compound Isoliquiritigenin Inhibits Breast Cancer Neoangiogenesis via VEGF/VEGFR-2 Signaling Pathway
Source: PLoS One. 2026 Feb 26;21(2):e0343779. doi: 10.1371/journal.pone.0343779 (PMC12944767; doi:10.1371/journal.pone.0343779)

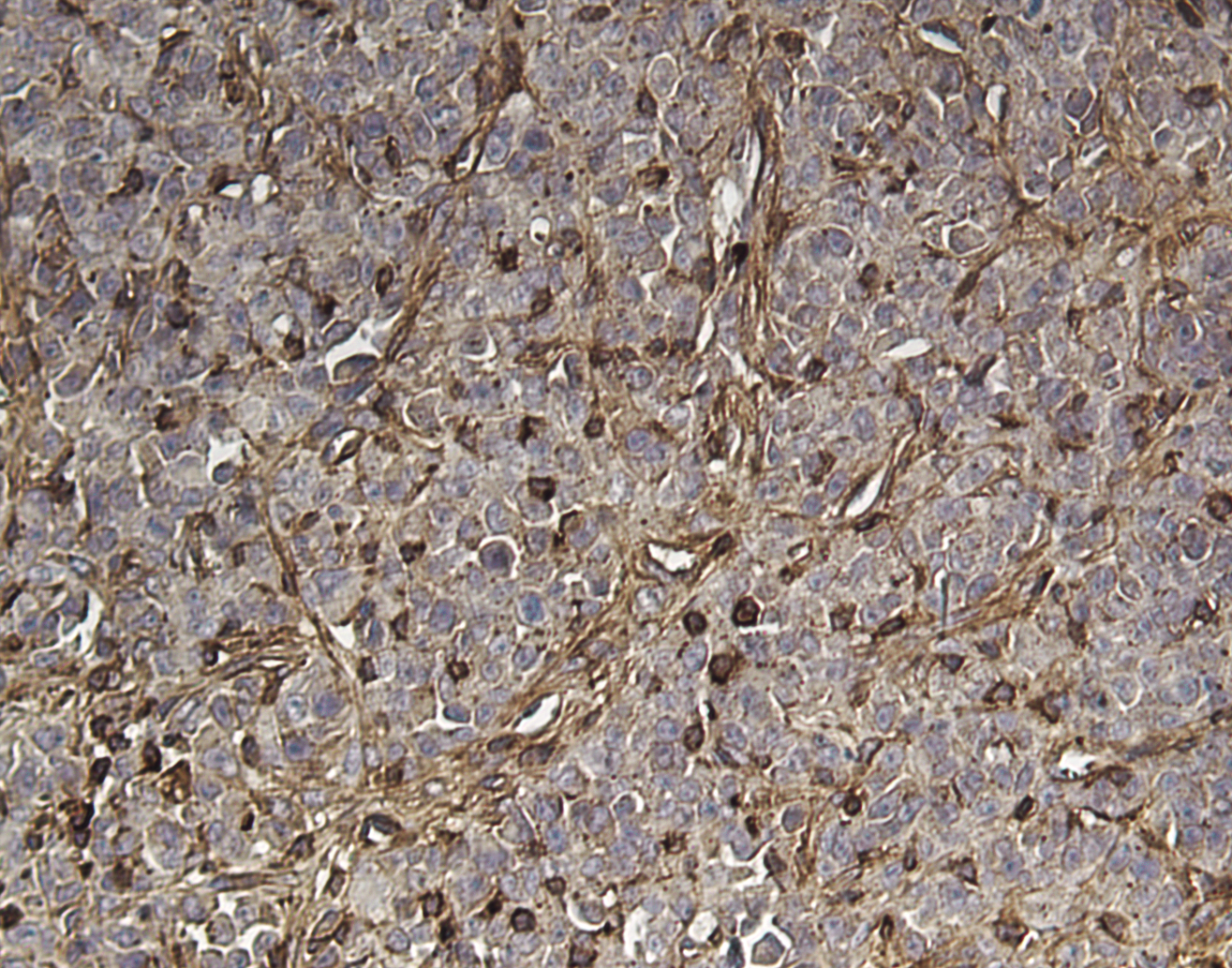

Supplement: S1 File — (ZIP) [file pone.0343779.s001.zip › S1 File/Representative images for Figure 6D/con-mmp2.tif]

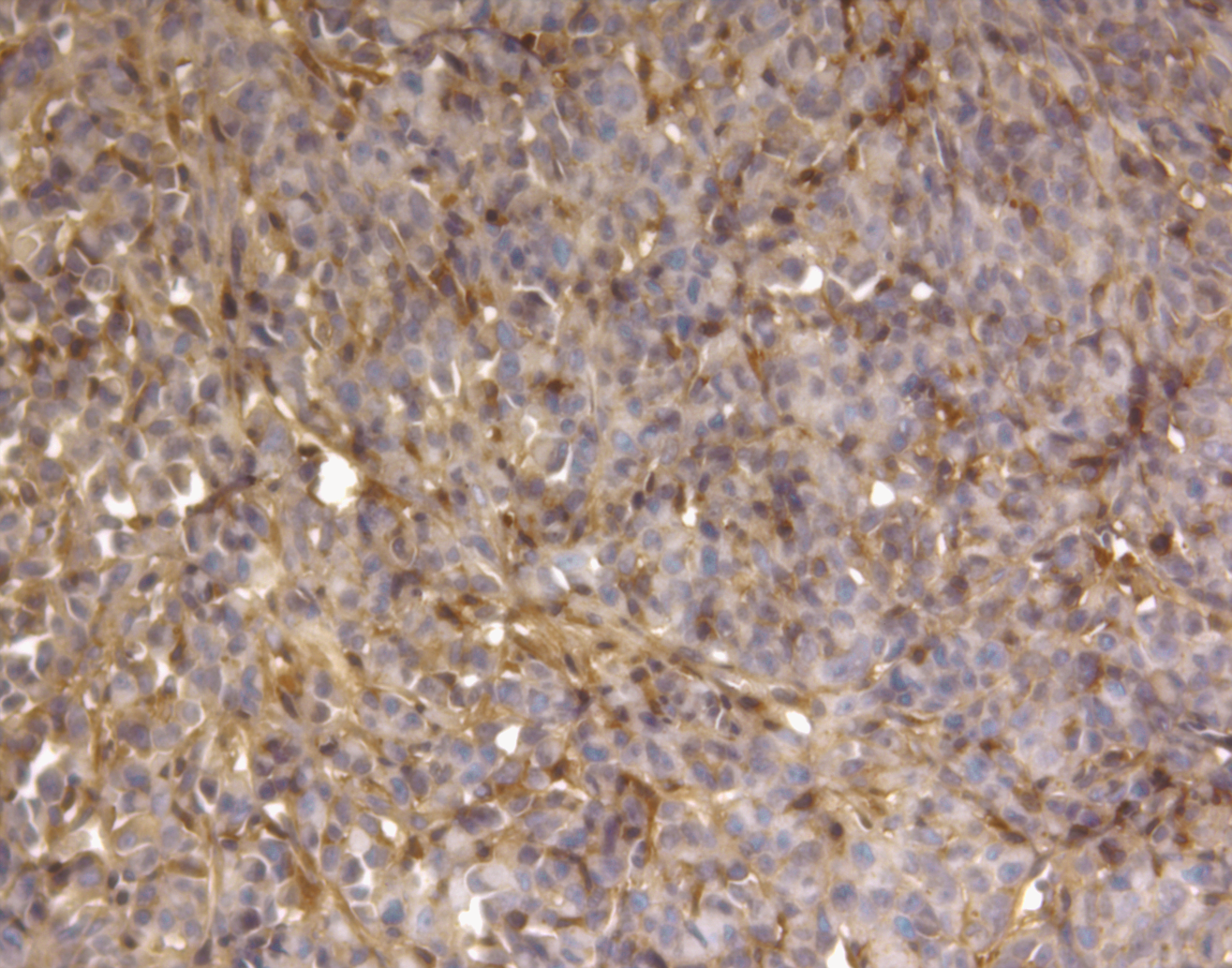

Supplement: S1 File — (ZIP) [file pone.0343779.s001.zip › S1 File/Representative images for Figure 6D/con-VEGFR2.tif]

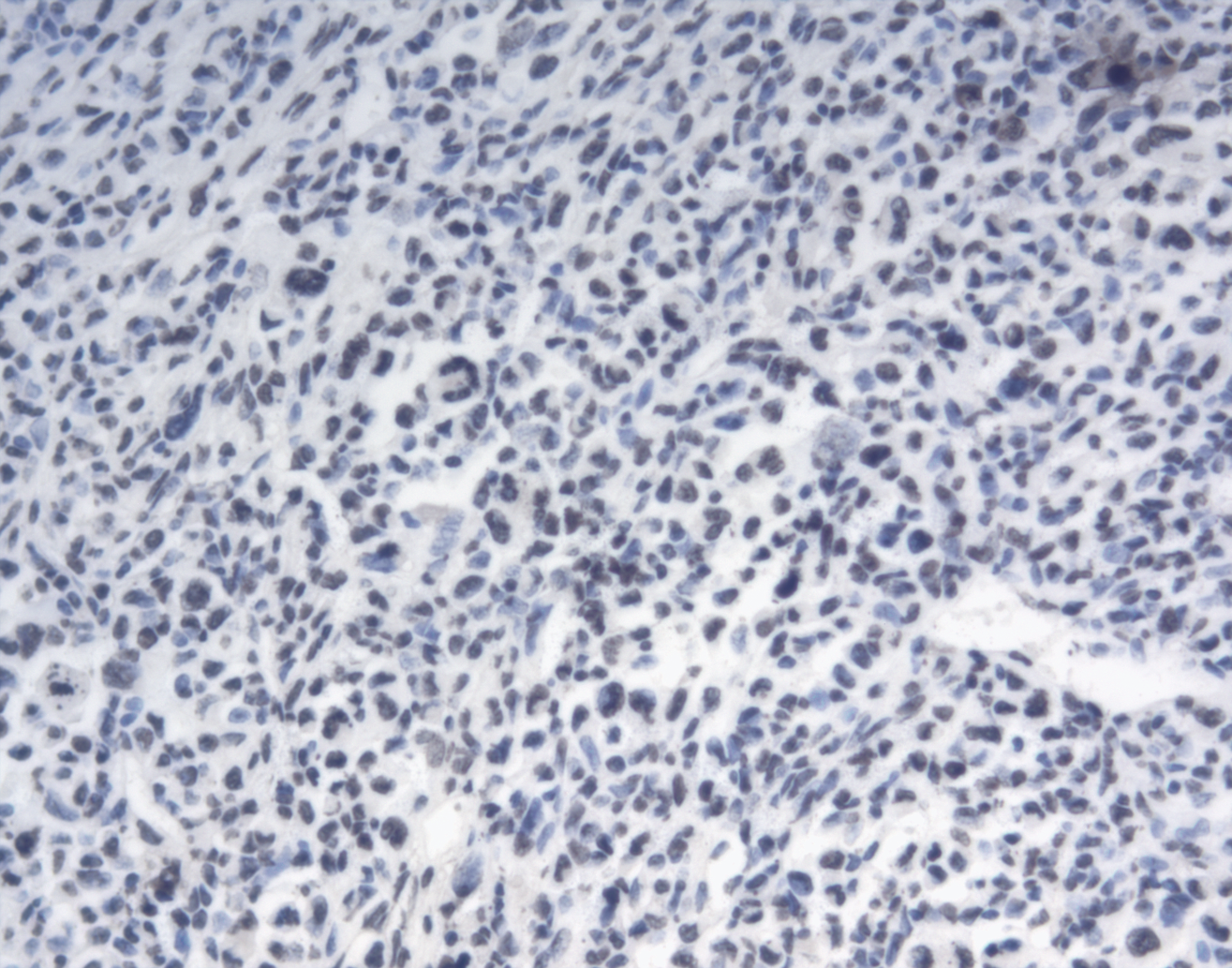

Supplement: S1 File — (ZIP) [file pone.0343779.s001.zip › S1 File/Representative images for Figure 6D/control TUNEL.tif]

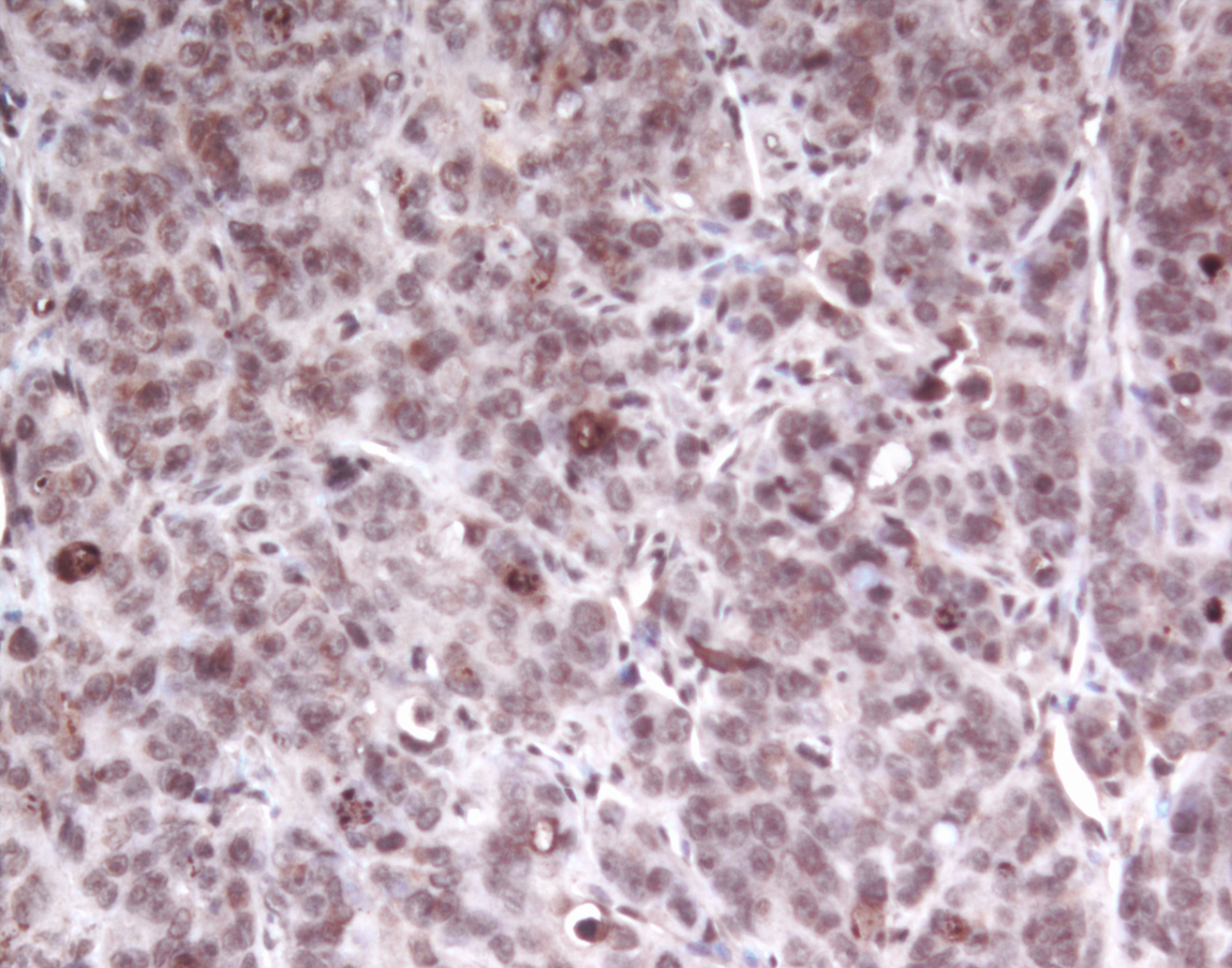

Supplement: S1 File — (ZIP) [file pone.0343779.s001.zip › S1 File/Representative images for Figure 6D/control VEGF.tif]

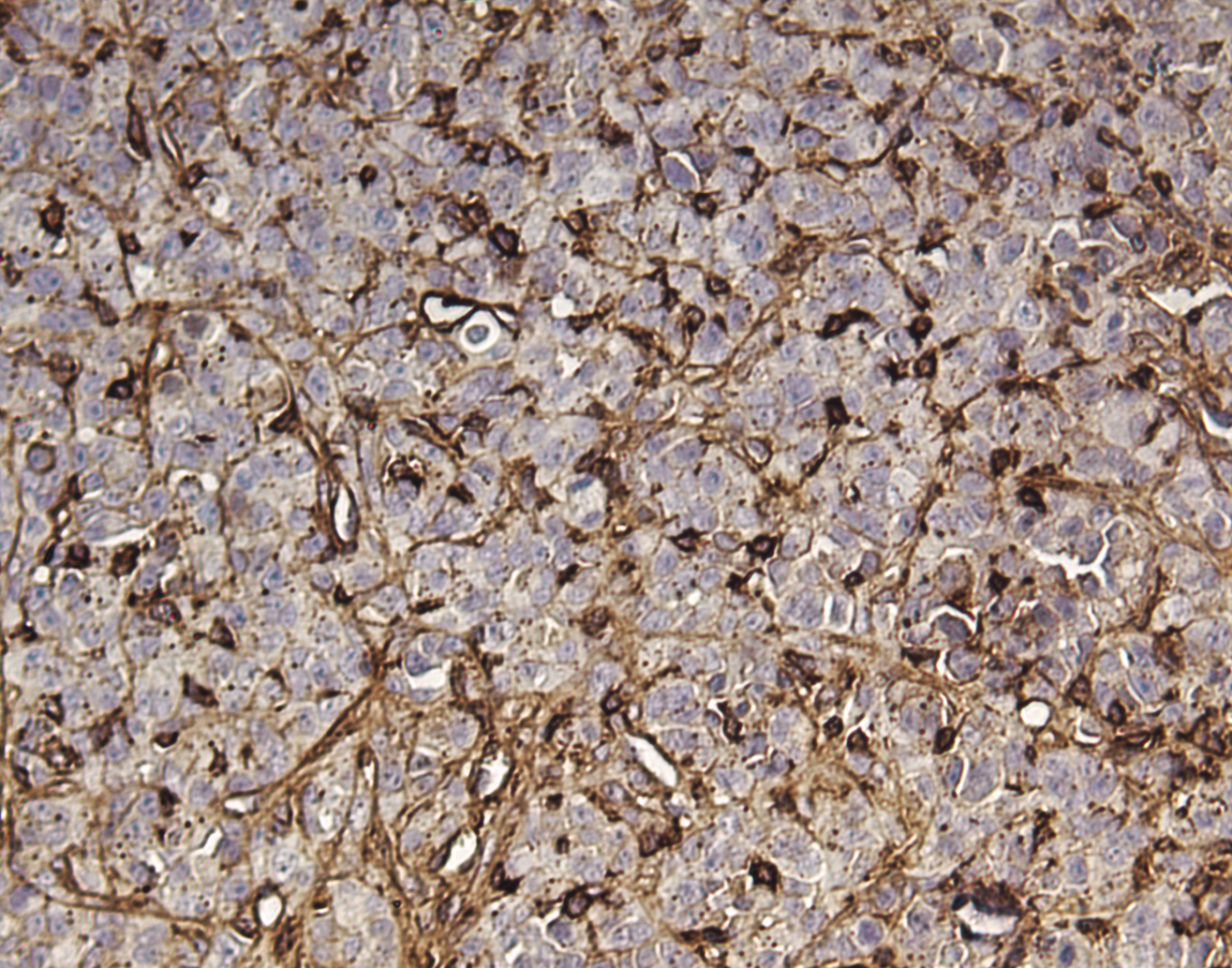

Supplement: S1 File — (ZIP) [file pone.0343779.s001.zip › S1 File/Representative images for Figure 6D/control-cd31.tif]

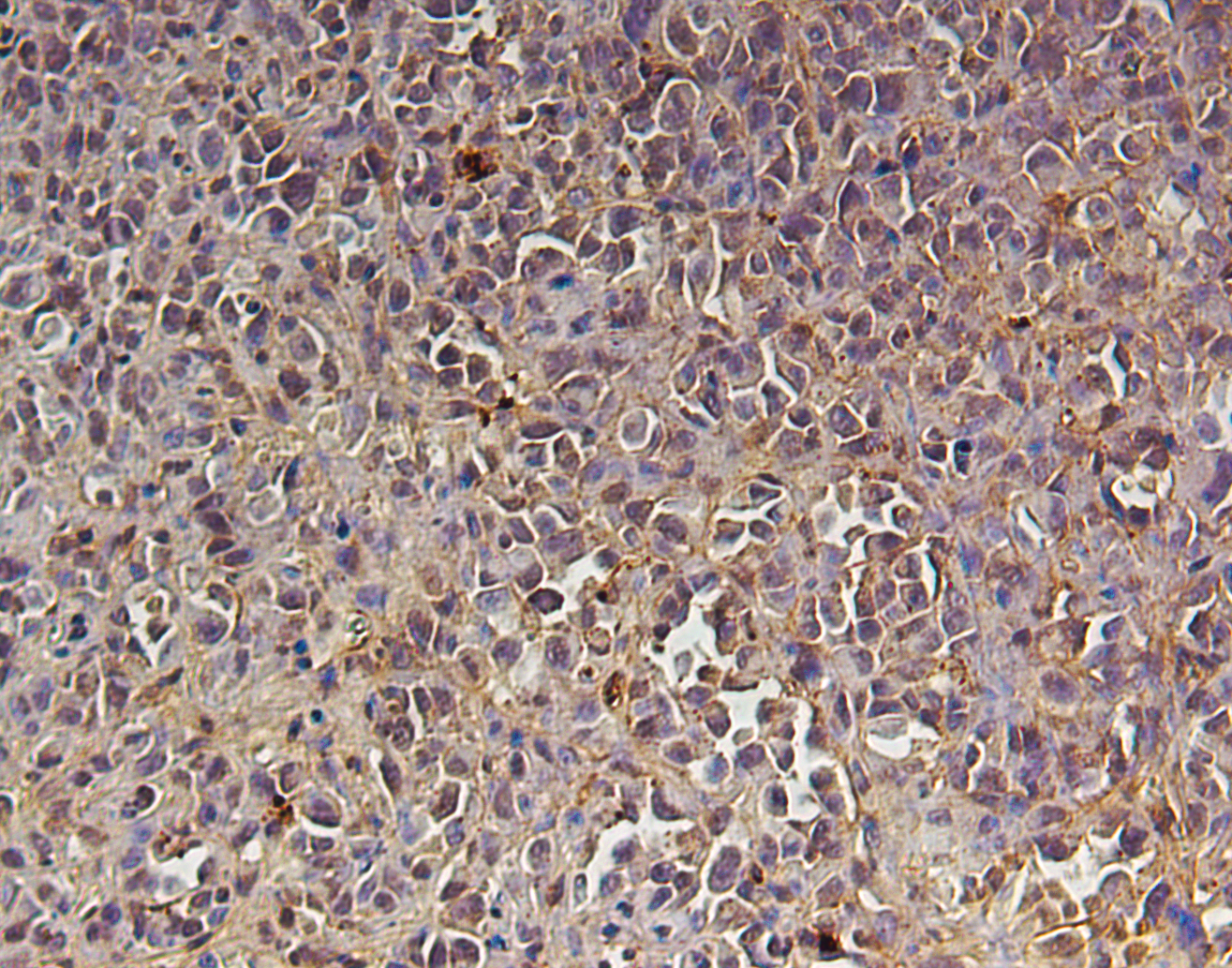

Supplement: S1 File — (ZIP) [file pone.0343779.s001.zip › S1 File/Representative images for Figure 6D/ISL CD31.tif]

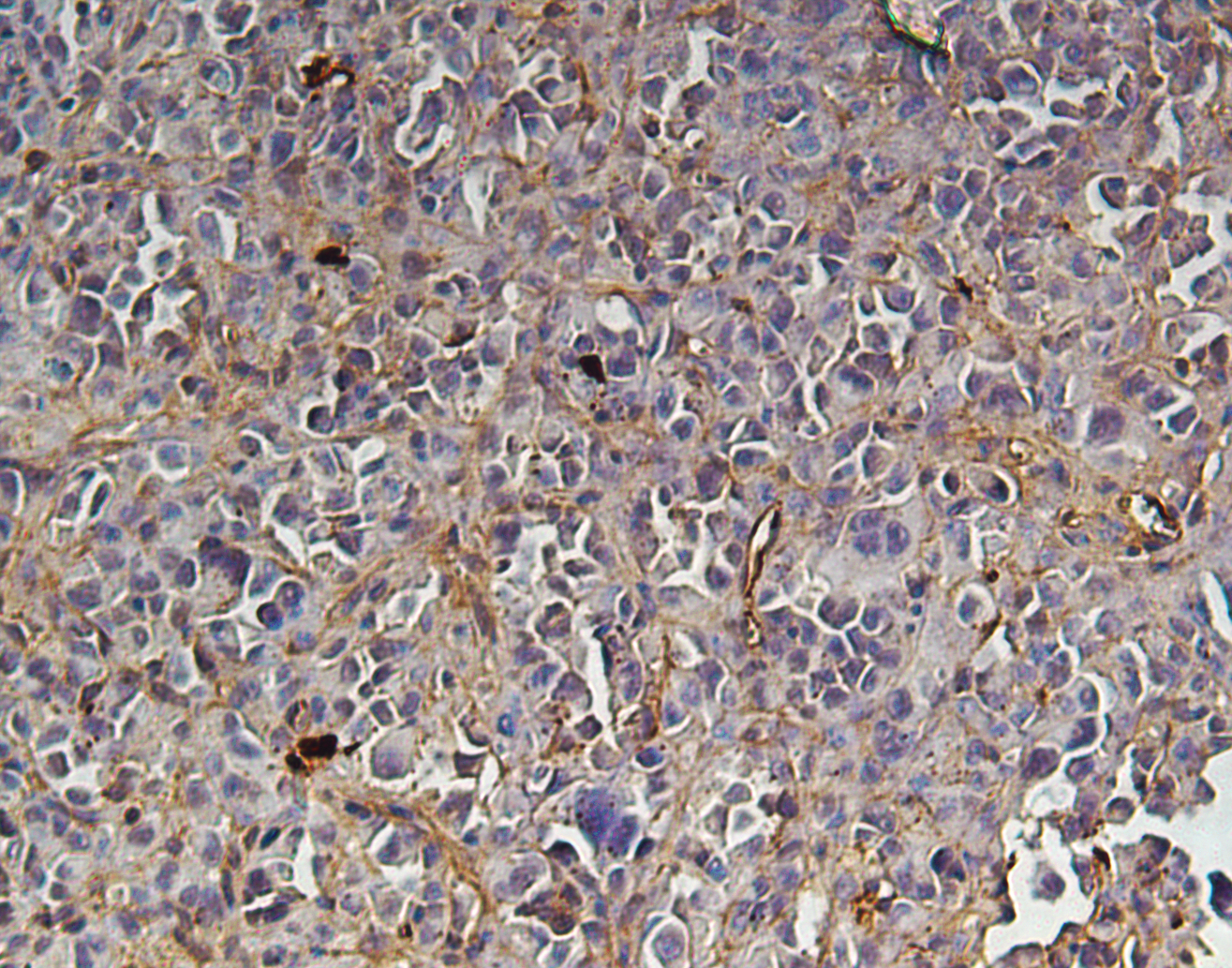

Supplement: S1 File — (ZIP) [file pone.0343779.s001.zip › S1 File/Representative images for Figure 6D/ISL MMP2.tif]

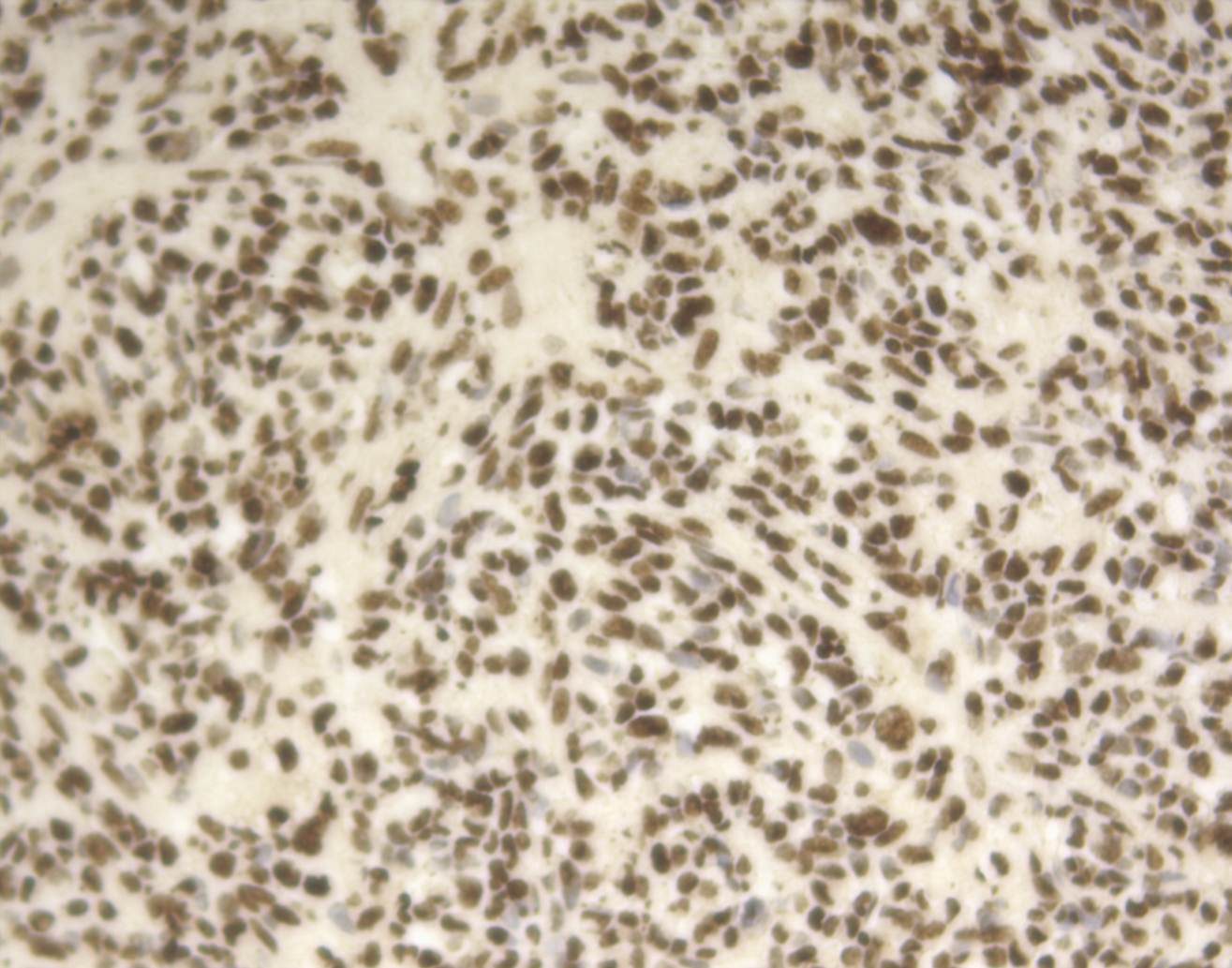

Supplement: S1 File — (ZIP) [file pone.0343779.s001.zip › S1 File/Representative images for Figure 6D/ISL TUNEL.tif]

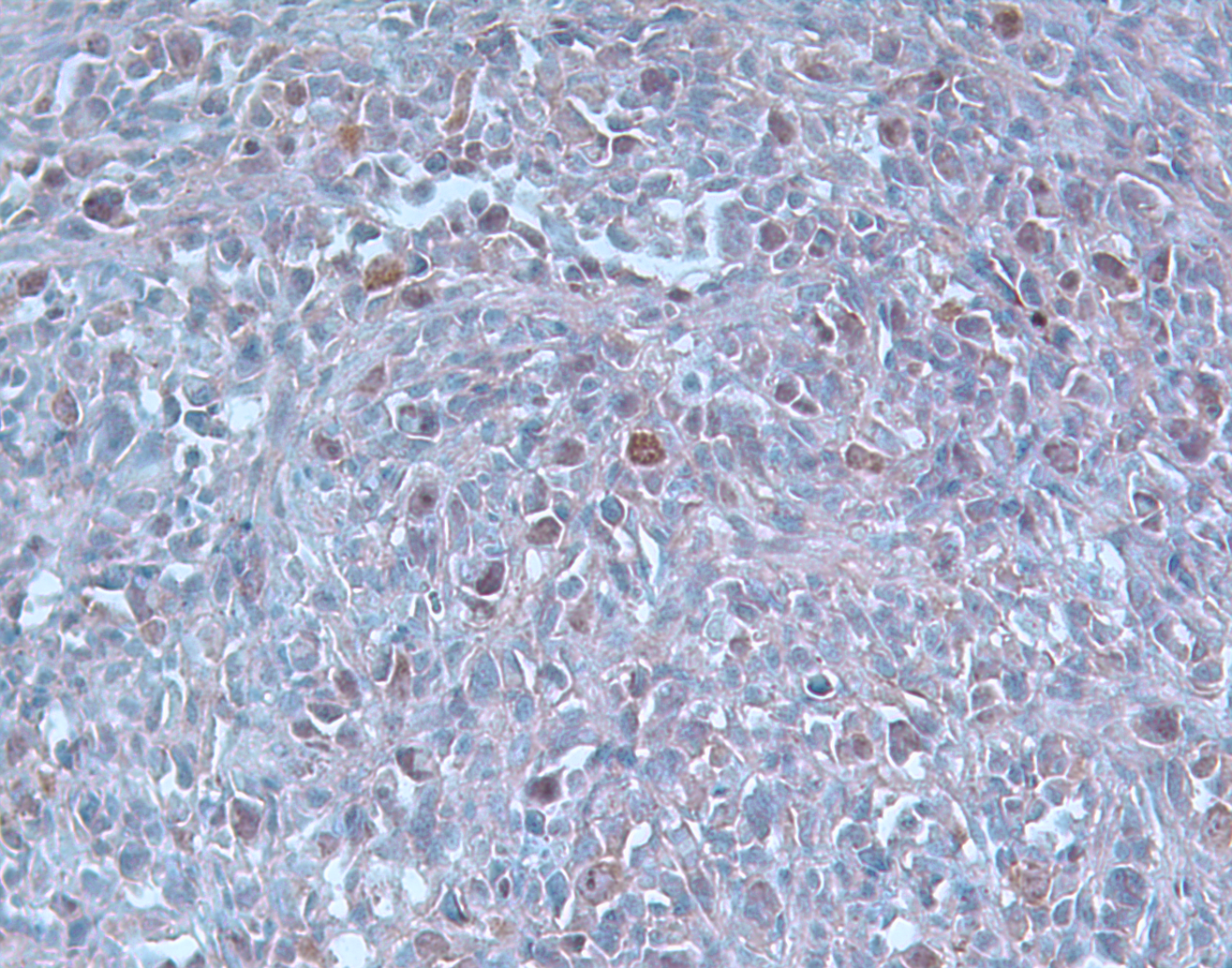

Supplement: S1 File — (ZIP) [file pone.0343779.s001.zip › S1 File/Representative images for Figure 6D/ISL VEGF.tif]

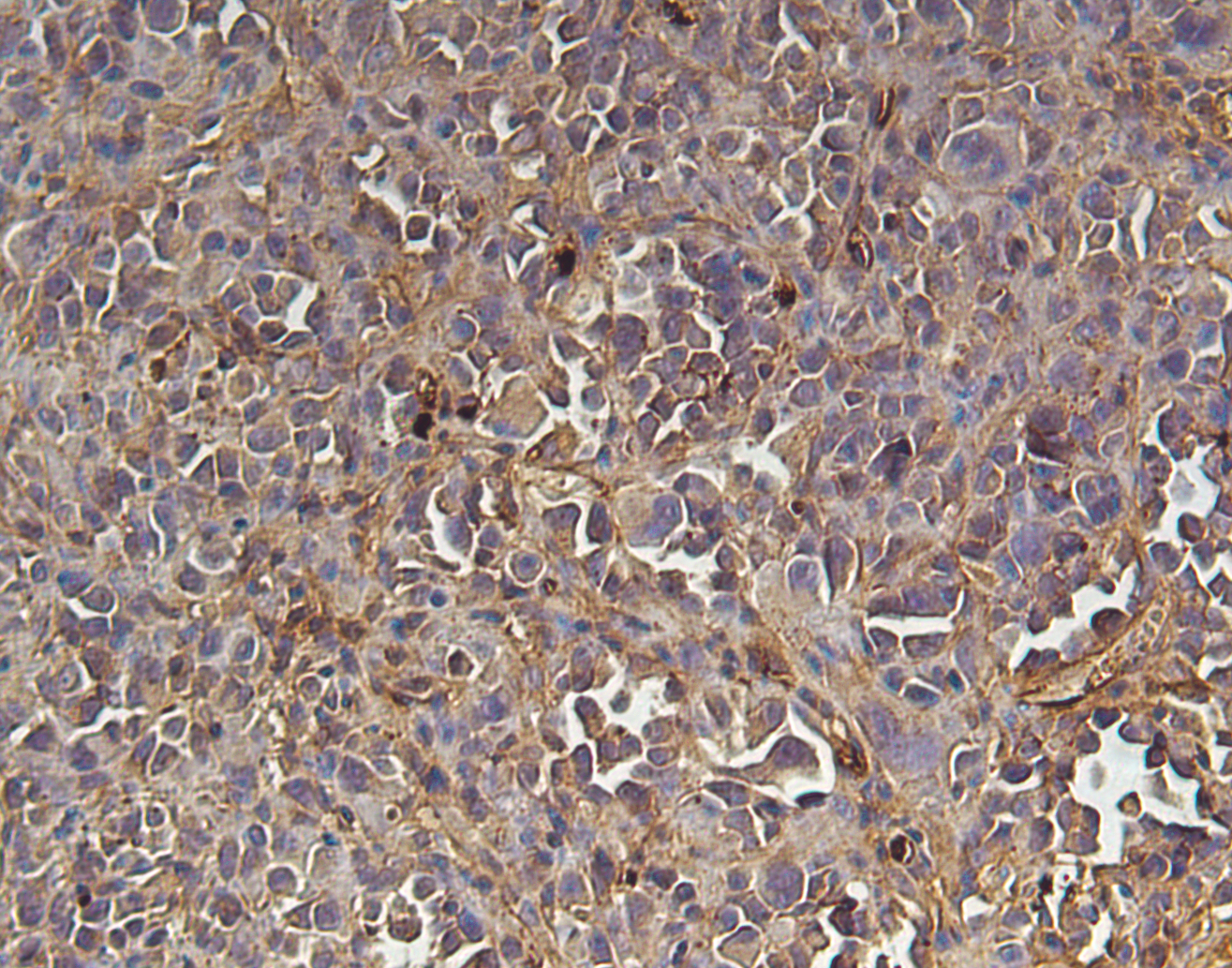

Supplement: S1 File — (ZIP) [file pone.0343779.s001.zip › S1 File/Representative images for Figure 6D/ISL VEGFR2.tif]

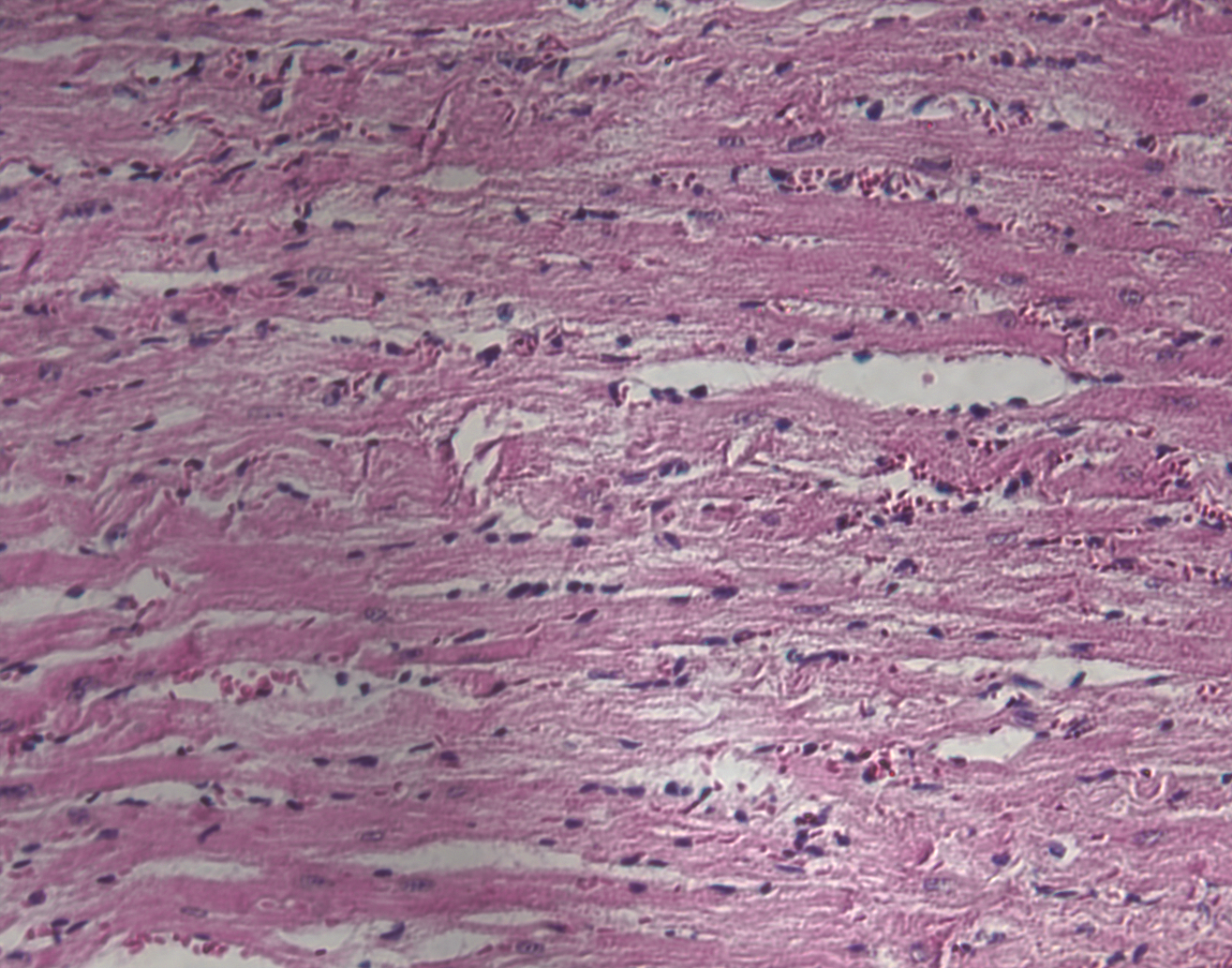

Supplement: S1 File — (ZIP) [file pone.0343779.s001.zip › S1 File/Representative images for Figure 6E/control heart.tif]

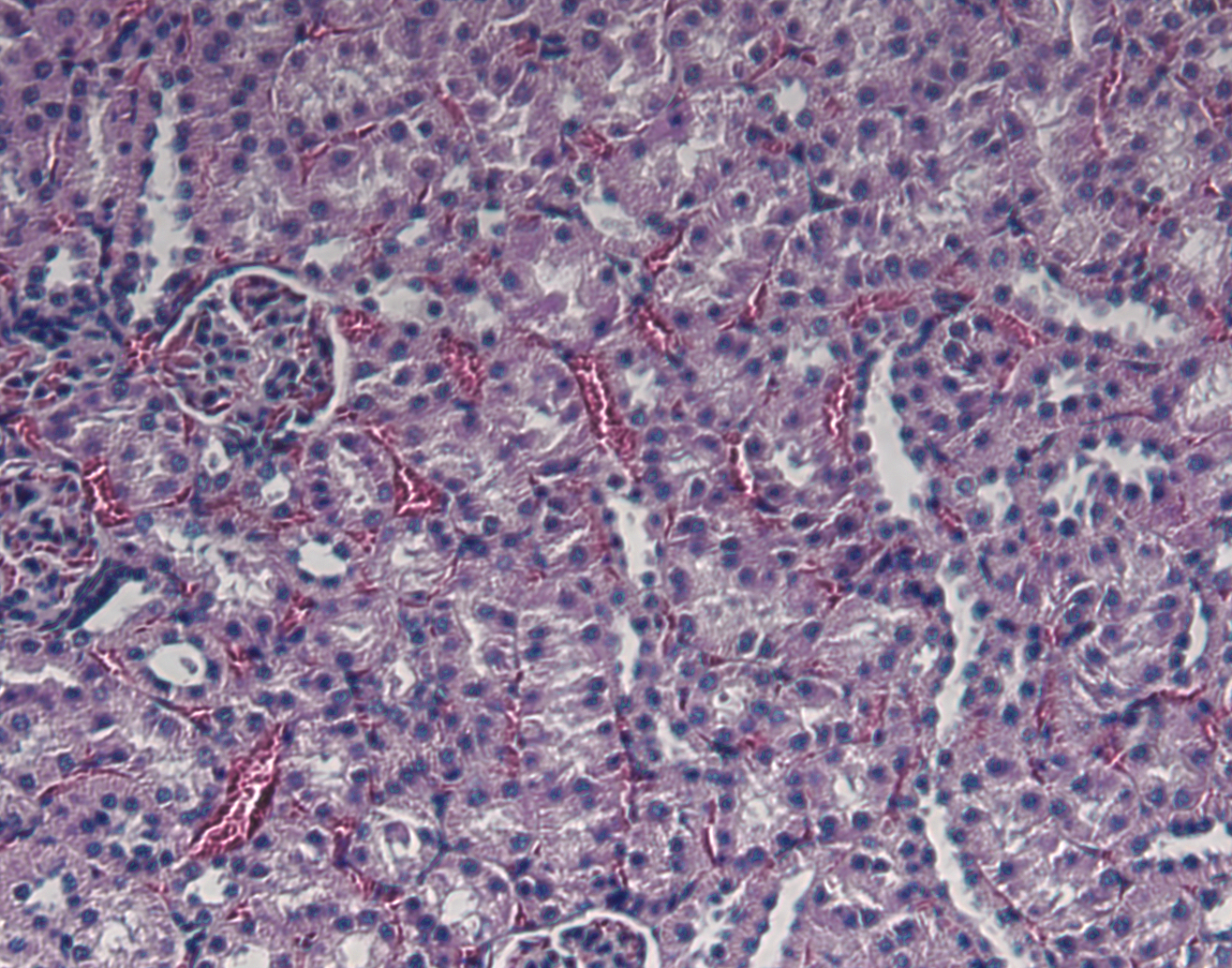

Supplement: S1 File — (ZIP) [file pone.0343779.s001.zip › S1 File/Representative images for Figure 6E/control kidney.tif]

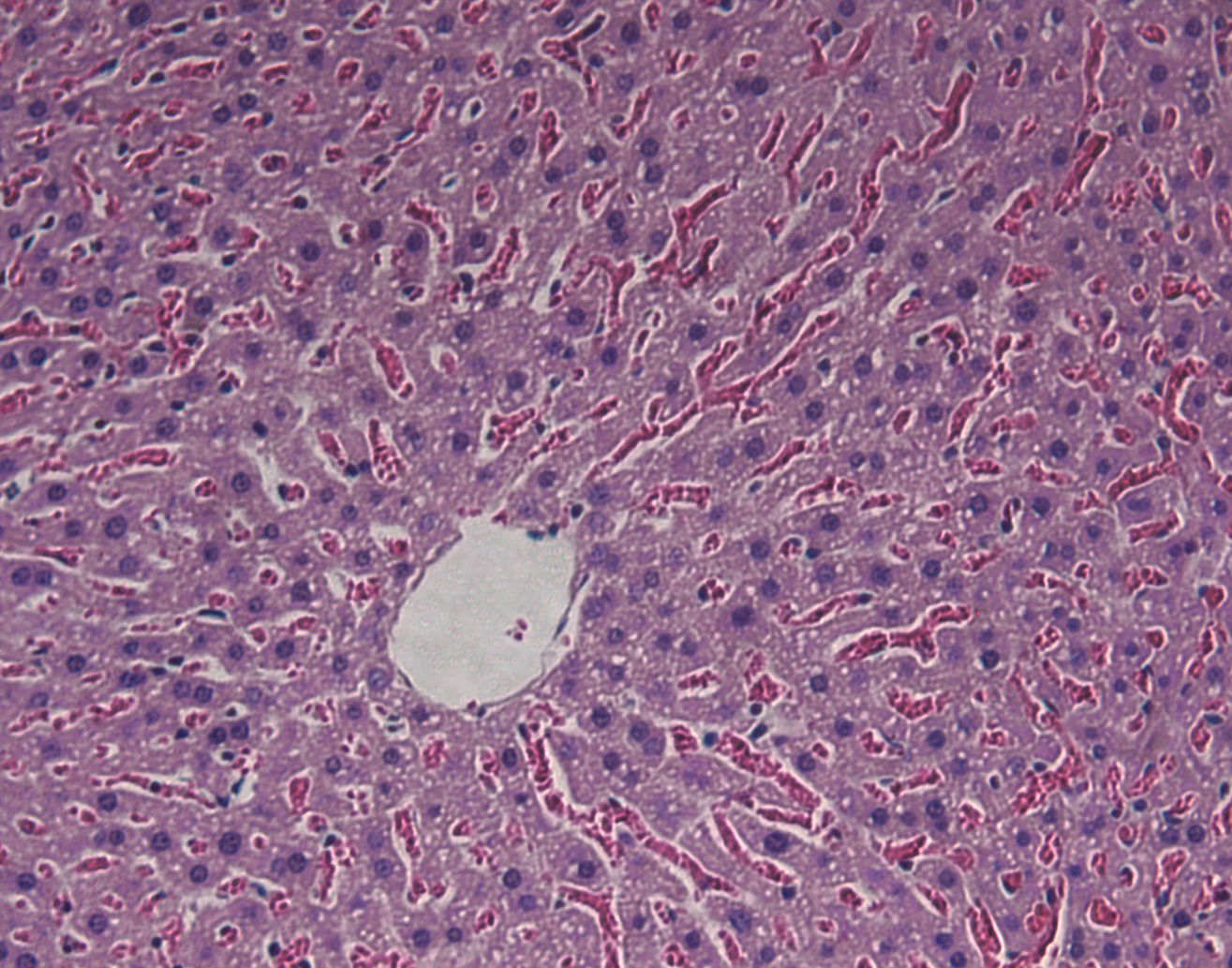

Supplement: S1 File — (ZIP) [file pone.0343779.s001.zip › S1 File/Representative images for Figure 6E/control liver.tif]

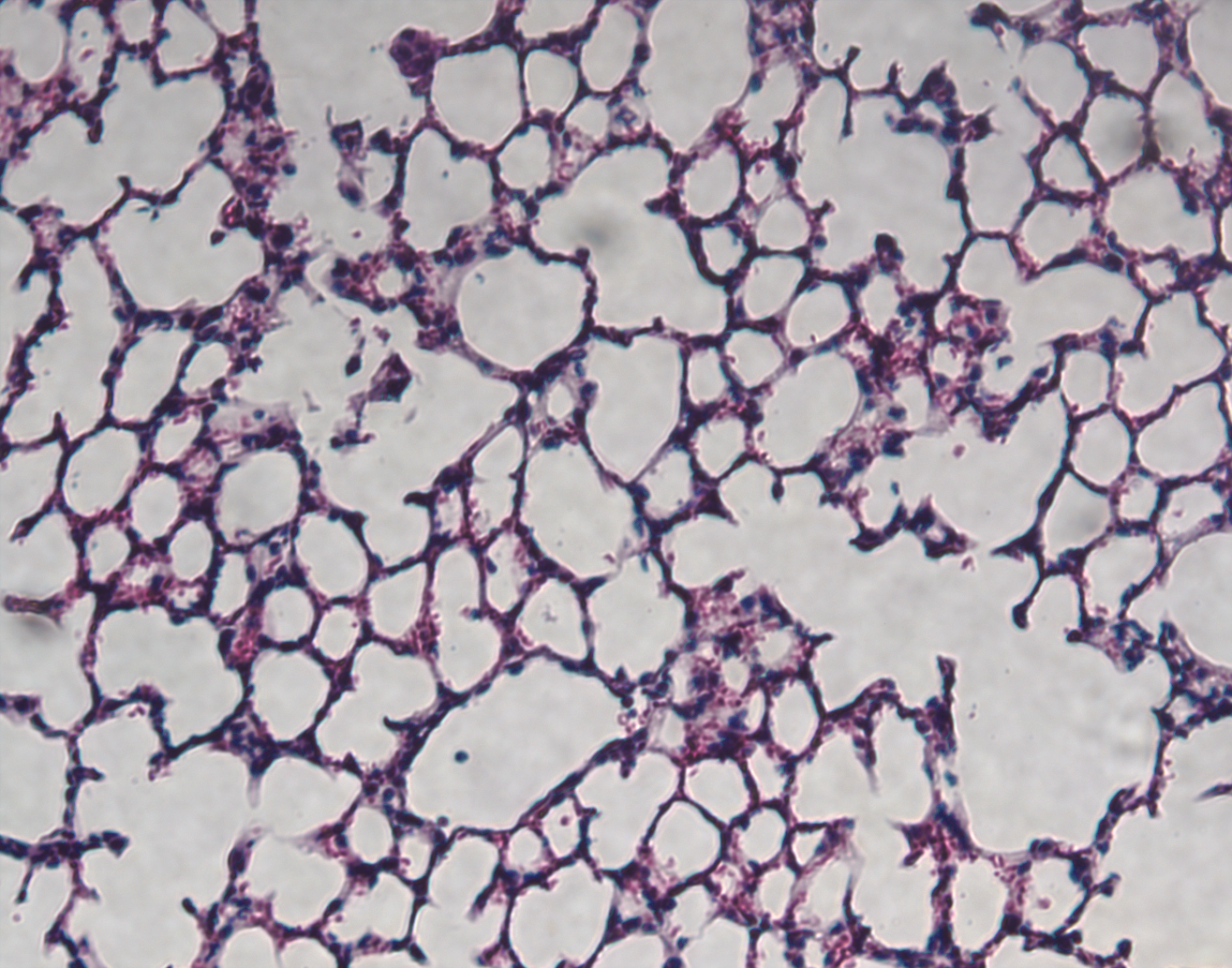

Supplement: S1 File — (ZIP) [file pone.0343779.s001.zip › S1 File/Representative images for Figure 6E/control lung.tif]

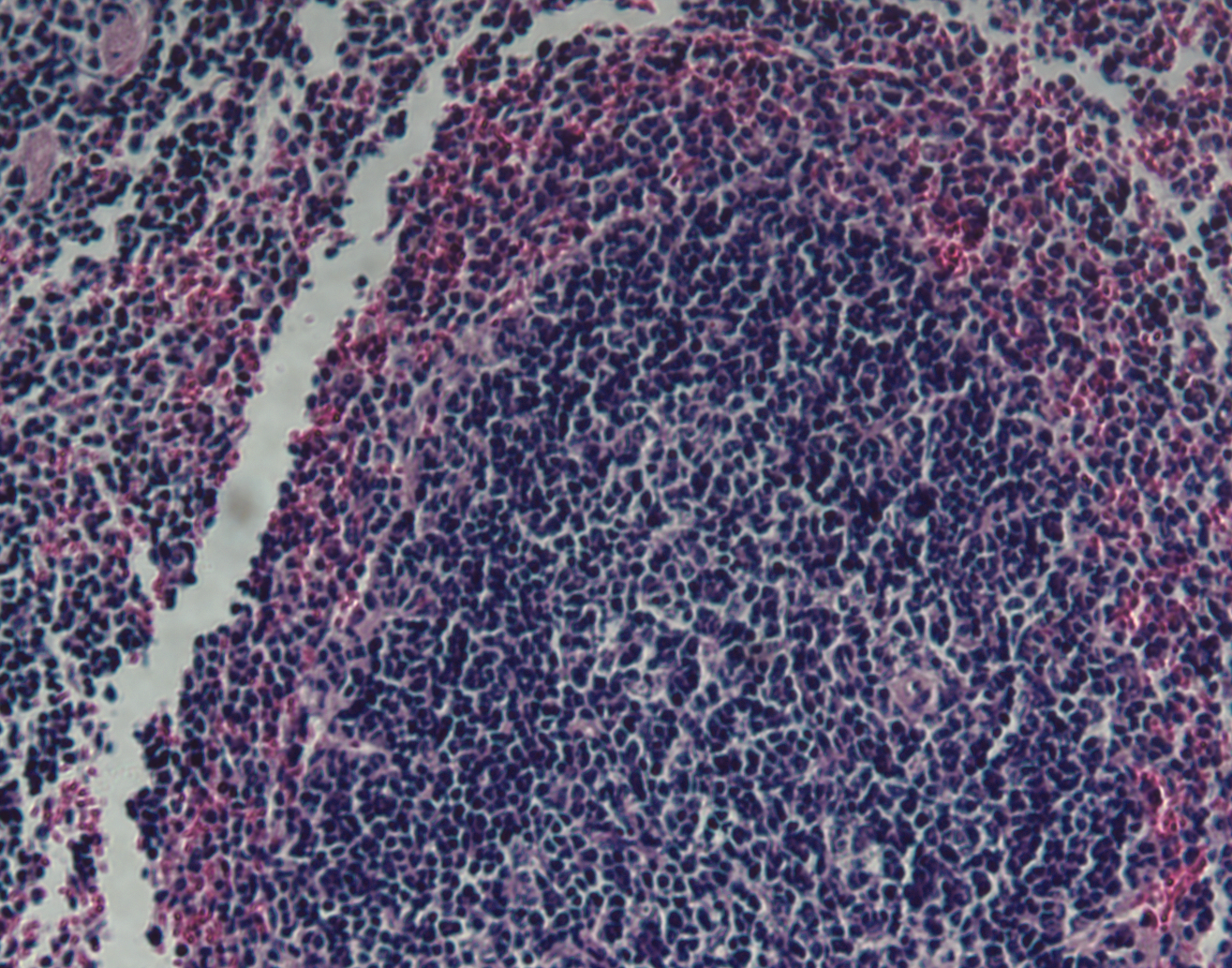

Supplement: S1 File — (ZIP) [file pone.0343779.s001.zip › S1 File/Representative images for Figure 6E/control spleen.tif]

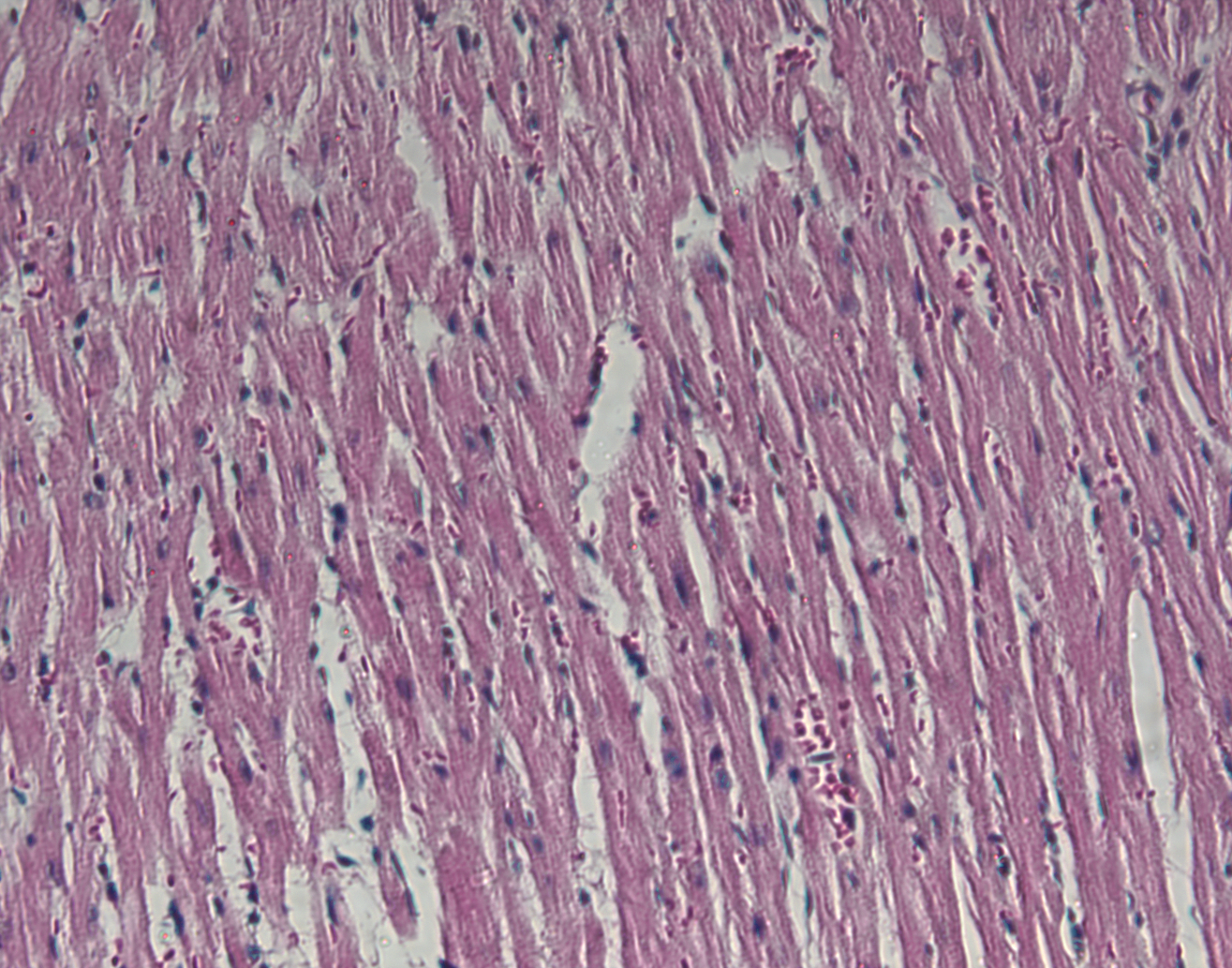

Supplement: S1 File — (ZIP) [file pone.0343779.s001.zip › S1 File/Representative images for Figure 6E/ISL heart.tif]

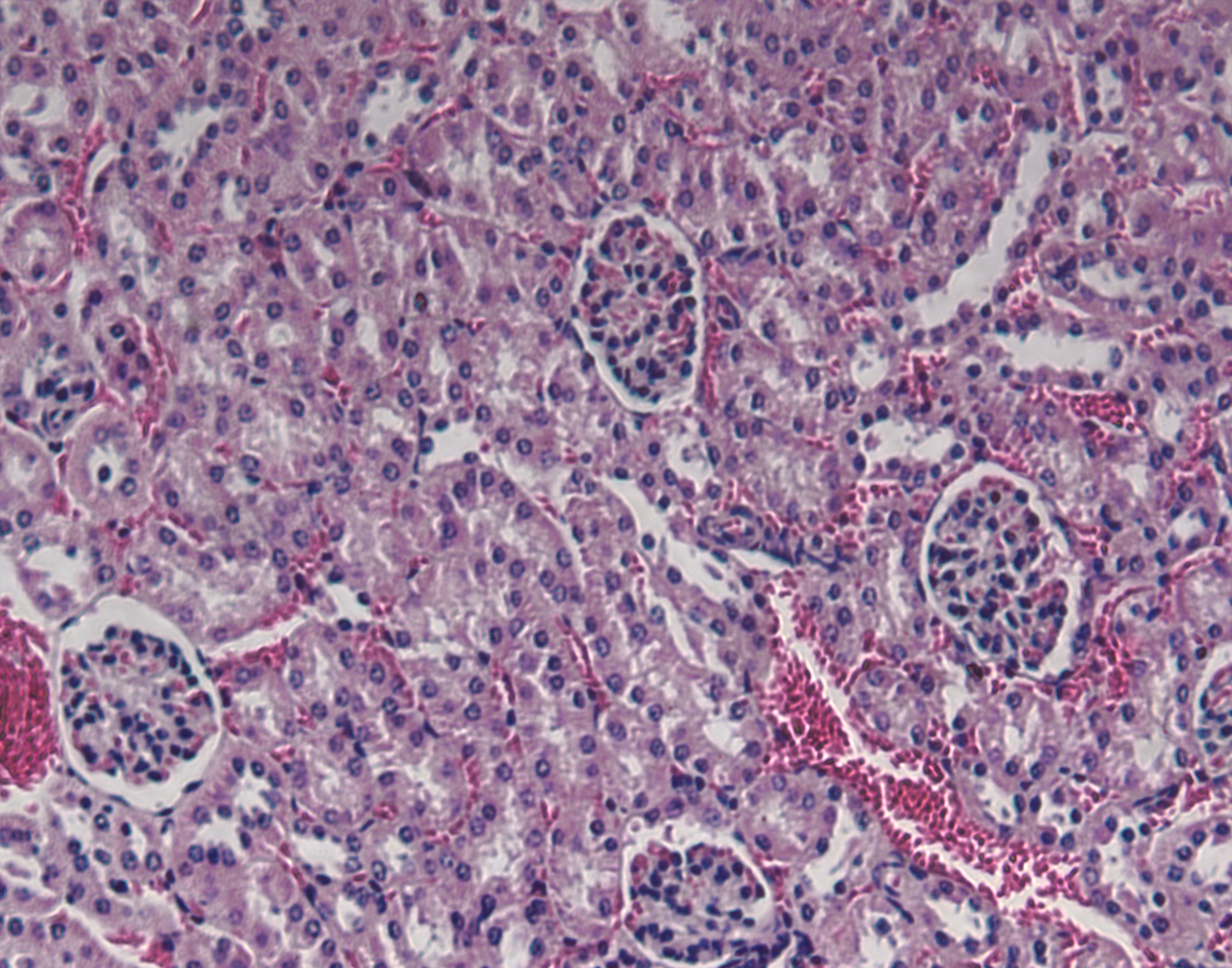

Supplement: S1 File — (ZIP) [file pone.0343779.s001.zip › S1 File/Representative images for Figure 6E/ISL kidney.tif]

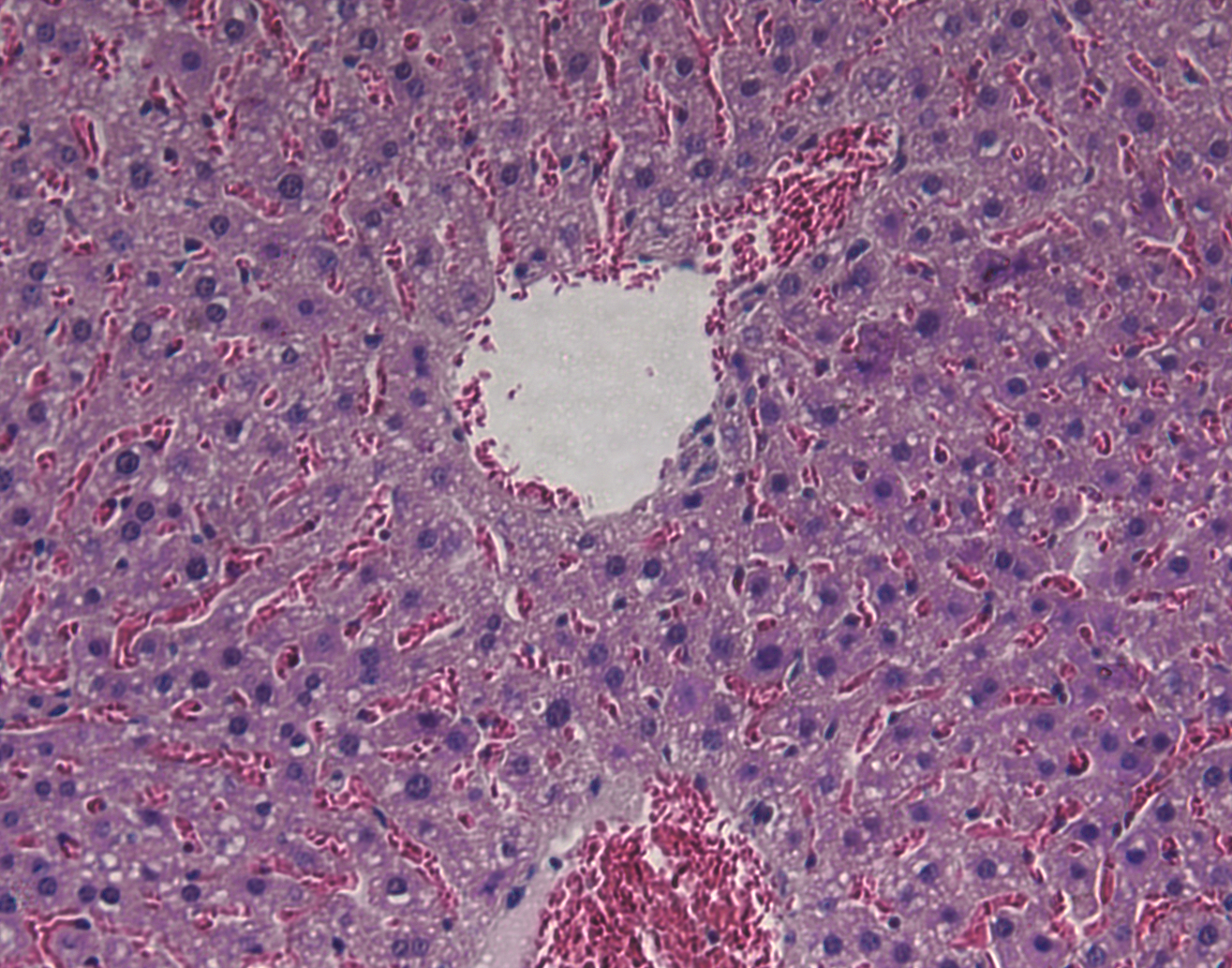

Supplement: S1 File — (ZIP) [file pone.0343779.s001.zip › S1 File/Representative images for Figure 6E/ISL liver.tif]

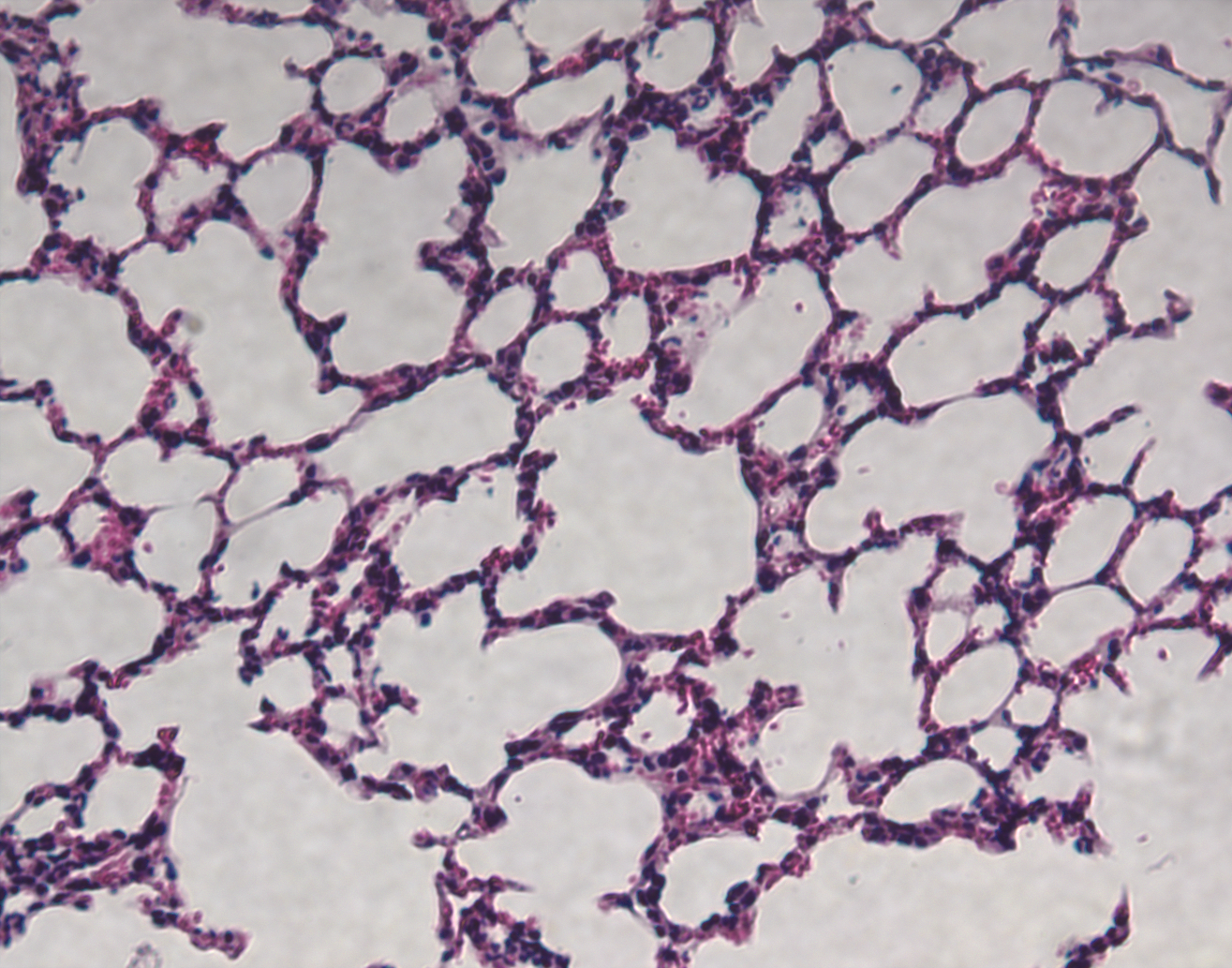

Supplement: S1 File — (ZIP) [file pone.0343779.s001.zip › S1 File/Representative images for Figure 6E/ISL lung.tif]

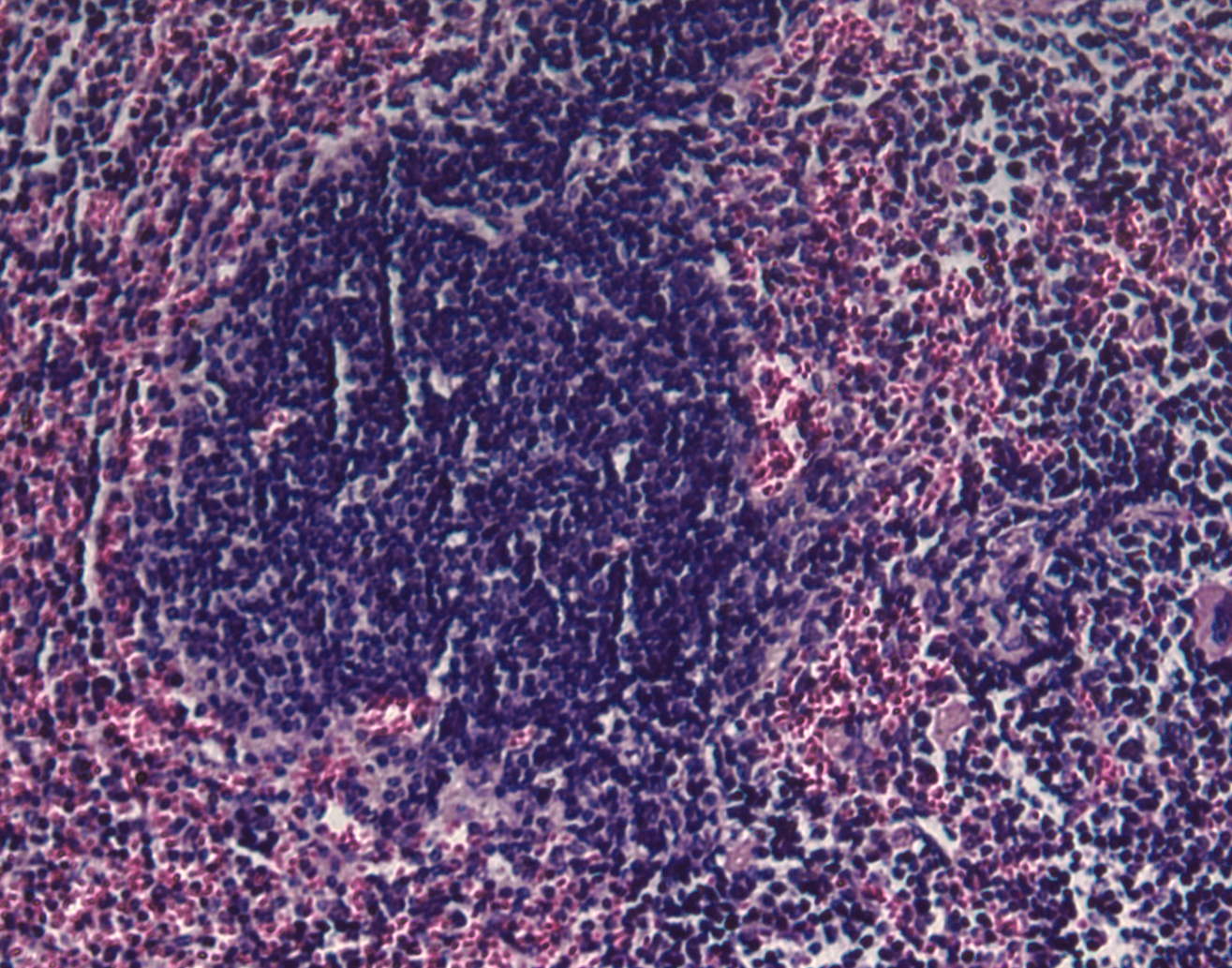

Supplement: S1 File — (ZIP) [file pone.0343779.s001.zip › S1 File/Representative images for Figure 6E/ISL spleen.tif]
